# Effects of growth rate, cell size, motion, and elemental stoichiometry on nutrient transport kinetics

**DOI:** 10.1371/journal.pcbi.1006118

**Published:** 2018-04-27

**Authors:** Kevin J. Flynn, David O. F. Skibinski, Christian Lindemann

**Affiliations:** 1 Biosciences, Swansea University, Swansea, United Kingdom; 2 Swansea University Medical School, Swansea University, Swansea, United Kingdom; 3 Department of Biological Sciences, University of Bergen, Bergen, Norway; University of California Irvine, UNITED STATES

## Abstract

Nutrient acquisition is a critical determinant for the competitive advantage for auto- and osmohetero- trophs alike. Nutrient limited growth is commonly described on a whole cell basis through reference to a maximum growth rate (*G*_*max*_) and a half-saturation constant (*K*_*G*_). This empirical application of a Michaelis-Menten like description ignores the multiple underlying feedbacks between physiology contributing to growth, cell size, elemental stoichiometry and cell motion. Here we explore these relationships with reference to the kinetics of the nutrient transporter protein, the transporter rate density at the cell surface (*TRD*; potential transport rate per unit plasma-membrane area), and diffusion gradients. While the half saturation value for the limiting nutrient increases rapidly with cell size, significant mitigation is afforded by cell motion (swimming or sedimentation), and by decreasing the cellular carbon density. There is thus potential for high vacuolation and high sedimentation rates in diatoms to significantly decrease *K*_*G*_ and increase species competitive advantage. Our results also suggest that *G*_*max*_ for larger non-diatom protists may be constrained by rates of nutrient transport. For a given carbon density, cell size and *TRD*, the value of *G*_*max*_*/K*_*G*_ remains constant. This implies that species or strains with a lower *G*_*max*_ might coincidentally have a competitive advantage under nutrient limited conditions as they also express lower values of *K*_*G*_. The ability of cells to modulate the *TRD* according to their nutritional status, and hence change the instantaneous maximum transport rate, has a very marked effect upon transport and growth kinetics. Analyses and dynamic models that do not consider such modulation will inevitably fail to properly reflect competitive advantage in nutrient acquisition. This has important implications for the accurate representation and predictive capabilities of model applications, in particular in a changing environment.

## Author summary

Relating environmental nutrient concentration and nutrient acquisition to cell growth is an important feature of numerical simulations describing ecological systems of microbes. Here we investigate the critical role of the combined effects of maximum growth rate, cell size, motion, and elemental stoichiometry on nutrient transport kinetics and thence growth kinetics. By applying mechanistic scaling of nutrient uptake our results identify fundamental shortcomings in the interpretation of empirically derived relationships used to describe nutrient uptake in microbes. While the amount of nutrient required to grow at a given rate under nutrient limited conditions increases rapidly with cell size, the maximum growth rate scales directly with the environmental nutrient concentration. Requiring less nutrient at lower maximum growth rates, cells can therefore remain healthier at lower resource abundance. Further, decreased carbon content per cell lowers demand for nutrient transport per surface area significantly. This allows larger phytoplankton cells, like diatoms, to significantly increase their competitive advantage with increasing sedimentation rates. These findings have important implications for numerical models both in a context of theoretical ecology and applied science. Our results highlight the importance of accounting for organism physiology and related feedbacks in ecological applications and climate change studies.

## Introduction

The relationship between nutrient uptake kinetics and growth rate is seen as a critical determinate in competition for organisms reliant on the transport of dissolved nutrients, and often plays a key role in structuring marine ecosystem models [1–3]. Here we consider interactions between cell size and cellular carbon density (as linked to vacuolation, for example), elemental stoichiometry, motion through the water, and growth rate potential with nutrient transport. While facets of such interactions have been considered before [3–5] we present a new analysis that explores how traits at the level of nutrient transport work through to better explain how nutrient availability controls organism growth and competitive advantage.

The physiology underpinning these relationships is complex and there is scope for significant confusion in interpreting experiment design and data. Most obviously there is the difference between the short term relationship between nutrient (substrate) concentration at the cell surface (*S*_*0*_) and nutrient transport rate into the organism, and the longer term relationship between *S*_*0*_ and organism growth rate. This difference develops because nutrient transport is controlled by various feedback processes that develop during post-transport assimilation of the nutrient, and are thus related to the organisms’ physiological history and thence to its growth rate. These factors also affect the difference between *S*_*0*_ and the substrate concentration in the bulk water (S_*∞*_); it is the latter which is determined in chemical analyses of water and features as a variable in models, while the former is the concentration of importance for the organism itself.

Flynn (1998) [6] differentiated between transport and growth kinetics, noting that experiment design (especially with respect to the period of incubation and the type of nutrient) and the prior physiological history of the organism govern whether measured “uptake kinetics” are more in keeping with true transport kinetics or with growth kinetics [7,6]. To measure transport kinetics requires very short incubations (durations of seconds) or extrapolation of time course incubations [6]. However, for practical reasons experiments are typically run over times from a few minutes up to several hours which is sufficient for the development of some level of satiation feedback that moderates the transport process. That incubation period is also usually insufficient to allow nutrient flow through to growth to approach steady-state. In consequence, interpreting reports in the literature concerning nutrient uptake kinetics conducted on different organisms, using different experimental protocols, is fraught with difficulties.

It is often assumed that for transport, uptake and growth kinetics the relationship with the substrate may be described using a rectangular hyperbolic type 2 (RHt2) function. RHt2 describes the process rate (*V*) as limited to a maximum rate, (*V*_*max*_) and with a half saturation constant (*K*) of the substrate concentration (*S*).

$$V=V_{max}\frac{S}{S+K}$$ (1)

With *K* usually written as *K*_*M*_, Eq 1 describes the Michaelis-Menten equation for enzyme kinetics. An analogous equation is used to describe Monod growth kinetics. To enable us to differentiate between transport, growth and uptake kinetics, we use terminologies analogous to those of Flynn (1998) [6]. Thus, with reference to the form of Eq 1, we differentiate between pairs of constants for maximum rate and *K*, respectively, controlling transport (*T*_*max*_ & *K*_*T*_), uptake (*U*_*max*_ & *K*_*U*_,) or growth (*G*_*max*_ & *K*_*G*_). Table 1 gives a description of all abbreviations used in this work.

**Table 1:** Description of variables.

Variable	Unit	Description
*C*_*cell*_	pgC cell^-1^	Cellular carbon content
*C*_*150*_	gC (L cell)^-1^	Cell with a fixed cellular carbon density of 150gC L^-1^ and, where appropriate, mobility related to *ESD* using Eq 12.
*C*_*prot*_	gC (L cell)^-1^	Cell representing a generic protist phytoplankton, where the cellular carbon density is allometrically scaled according to [9] and, where appropriate, mobility is related to *ESD* using Eq 12.
*C*_*diat*_	gC (L cell)^-1^	Cell representing a diatom, where the cellular carbon density is allometrically scaled according to [9] and, where appropriate, sedimentation is related to *ESD* using Eq 13.
*ESD*	μm	Cell equivalent spherical diameter
*G*	d^-1^	Growth rate limited by *G*_*max*_
*G*_*max*_	d^-1^	Maximum growth rate
*G*_*0*.*5*_	d^-1^	*G* = *G*_*max*_/2, enabled when *S*_*0*_ = *K*_*G*_
*k*_*cat*_	mole-specific rate (s^-1^)	Turnover number for pure enzyme
*K*_*G*_^*1*^	substrate concentration in bulk medium (S_∞_)	Substrate concentration in the bulk medium supporting a growth rate of *G* = *G*_*max*_/2 (i.e., *G*_*0*.*5*_). This is not an input for a rectangular hyperbolic function, but an emergent value.
*K*_*M*_	substrate concentration at site of enzyme (S_0_)	Michaelis-Menten half saturation constant for the rectangular hyperbolic description of enzyme activity
*K*_*T*_	substrate concentration at site of transporter (S_0_)	Analogous to *K*_*M*_, but for substrate transporter operation to bring nutrient into a cell
*K*_*U*_	substrate concentration in bulk medium (S_∞_)	Experimentally derived half saturation constant for rectangular hyperbolic description of substrate uptake. For very small cells, and very short period experiments, *K*_*U*_ ≡ *K*_*T*_ (see [6]); this variable is termed “*K*” in [10,11].
*KQN*	Dimensionless	Constant for normalised quota-control of N-specific growth
*NC*	gN gC^-1^	N:C cell quota; for N-limited growth this varies between *NC*_*min*_ and *NC*_*max*_
*NC*_*max*_	gN gC^-1^	Maximum N:C cell quota for N-limited growth, at which *G* = *G*_*max*_
*NC*_*min*_	gN gC^-1^	Minimum N:C cell quota for N-limited growth, at which *G* = 0
*S*_*0*_	mol m^-3^	Substrate concentration at the site of the enzyme or transporter protein
*S*_*∞*_	mol m^-3^	Substrate concentration at (nominal) infinite distance from the enzyme or transporter protein. This is the bulk water substrate concentration.
*SA*	μm^2^	Cell surface area; 4π(*ESD*/2)^2^
*T*	substrate (cell)^-1^ time^-1^	Transport rate limited by *T*_*max*_ as a function of substrate concentration at the site of the transporter
*T*_*absmax*_	substrate (cell)^-1^ time^-1^	Absolute maximum value of *T*_*max*_; usually occurs under intermediate nutrient-stress.
*T*_*max*_	substrate (cell)^-1^ time^-1^	Analogous to *V*_*max*_, but reporting the maximum rate of substrate transport into a cell. This value varies with cell nutrient status, between a value of 0 and *T*_*absmax*_. *T*_*max*_ = *U*_*max*_ at time zero of the experimental incubation, prior to commencement of satiation feedback. See *K*_*U*_.
*TRD*	substrate transport time^-1^ μm^-2^	Transport Rate Density; substrate transported per unit of cell surface area. This aligns with the value of *T*_*max*_, expressed per unit of cell surface area.
*TRD*_*max*_	substrate transport time^-1^ μm^-2^	Maximum possible *TRD*. This aligns with the absolute maximum value of *T*_*absmax*_, expressed per unit of cell surface area.
*TRD*_*Gmax*_	substrate transport time^-1^ μm^-2^	Value of *TRD* required to enable transport at a rate commensurate with *G* = *G*_*max*_, which allows N:C = *NC*_*max*_
*U*	substrate (cell)^-1^ time^-1^	Uptake rate limited by *U*_*max*_
*U*_*max*_	substrate (cell)^-1^ time^-1^	Experimentally derived maximum substrate uptake rate. Under strictly controlled conditions, with very short period experiments, *U*_*max*_ ≡ *T*_*max*_ (see [6]); this variable is termed “*V*_*max*_” in [10,11].
*V*	substrate (g enzyme)^-1^ time^-1^	Enzyme activity limited by substrate availability to *V*_*max*_
*V*_*max*_	substrate (g enzyme)^-1^ time^-1^	Michaelis-Menten maximum enzyme activity
*δ*_*TRD*_	Dimensionless	*TRD*_*max*_: *TRD*_*Gmax*_; gives an index of over-capacity for transport.

^*1*^ Note that *K*_*G*_ is sometimes referred to as *K*_*s*_ in the literature. This notation can be somewhat misleading as *K*_*s*_ is traditionally used for the substrate half saturation content for a generic substrate-specific process.

In reality, as we shall see, the RHt2 curve may not always be appropriate for the task at hand. However, the reciprocal value of *K*, as the value of *S*_*0*_ at which *V* = *V*_*max*_/2 = *V*_0.5_, nonetheless provides an index for the relative affinity of the kinetics for a given value of *V*_*max*_. Ultimately, if all else is equal, an organism which requires a lower substrate concentration to support a growth rate (*G*) at half that of its maximum (i.e., *G* = *G*_*max*_/2 = *G*_0.5_) and thus expresses a lower *K*_*G*_, will be at an advantage over an organism with a higher *K*_*G*_. While in models *K*_*G*_ is usually set as an input constant, the real value is an emergent function of nutrient transport and whole organism physiology. For example, *K*_*G*_ for iron-limited phytoplankton growth depends greatly on whether nitrate or ammonium is used as the N-source, and also on the incident irradiance under which the phytoplankton grow [8]. To make the linkage between transport and growth kinetics thus requires an appreciation of the underlying physiology.

### Nutrient transport kinetics

Nutrient transport (e.g., of NO_3_^-^, NH_4_^+^, PO_4_^3-^) typically occurs via secondary active porters that are either matched for a specific nutrient molecule type, or for similar types [12]; thus a transporter for NO_3_^-^ will not transport NH_4_^+^, while similar amino acids such as the cationic group arginine, lysine, histidine and ornithine may share the same transporter [13]. In addition, individual nutrient types may be taken up by several different transporter proteins [14–16], some of which may support biphasic kinetics [16–18]. Here, to simplify discussions, we will consider transport via a single (monophasic) transporter type.

While transporter proteins are not strictly enzymes (as they typically do not change the chemical form of their substrate), they express an affinity for the nutrients they transport; by analogy with the Michaelis-Menten half saturation value of enzymes, *K*_*M*_, we term this substrate concentration *K*_*T*_. The constant *K*_*M*_ is a function of the affinity of the enzyme for the substrate in classic Michaelis-Menten terminology and is determined assuming that all factors other than substrate availability are non-limiting. Determining *K*_*T*_ is more complex because transporter functionality depends on the integrity of the membrane in which the transporter proteins function, ionic gradients generated by primary active transporters required to support the operation of the typically secondary-active nutrient-transporters, as well as on the aforementioned absence or presence of short and longer term feedback processes modulating transport itself into the functional cell.

Another defining criterion for enzyme functionality is the maximum level of activity, *k*_*cat*_, which is described in units of mole of substrate consumed (or product given) per mole of enzyme per unit of time (Table 1). The maximum rate of enzyme activity in a given sample of biological material, which is a product of *k*_*cat*_ and the concentration of enzyme protein, sets the value of the maximum process rate, *V*_*max*_, in Michaelis-Menten kinetics. It is important to note that the amount of enzyme in an assay does not affect the value of *K*_*M*_, while the value of *V*_*max*_ in the assay is linearly related to enzyme concentration. The value of *V*_*max*_ can thus be seen as being somewhat ambiguous, only being useful for a specific assay incubation. For considerations of whole-organism physiology, the value of *k*_*cat*_ needs to be placed in the context of the total demand for its activity, the size (mass) of the enzyme and thence for the total resource expenditure for that enzyme within a given cell (e.g., for such calculations applied to the enzyme fixing CO_2_, RuBisCO [19]).

The maximum rate of activity in a given cellular system (*T*_*max*_) is analogous to *V*_*max*_ in an enzyme assay. Accordingly, while the value of *K*_*T*_ is independent of the number of transporter proteins in the cell, the value of *T*_*max*_ is indeed dependent on that number. The extent to which *T*_*max*_ exceeds *G*_*max*_, noting that transporter activity is modulated by post-transport physiology, helps to explain why *K*_*G*_ is lower than *K*_*T*_, as illustrated in S1 Fig and the adjoining online text. In reality there are many hundreds if not thousands of transporter proteins in operation across the plasma-membrane of an individual cell. Theoretical estimates of relative nitrate and phosphate transporter density suggest that a specific transporter type will generally cover less than 0.1% of the cell surface under nutrient limited conditions [20]. The number of transporter proteins, and hence the maximum rate of nutrient transport (*T*_*max*_), also varies greatly with the nutritional status of the cell and for different nutrients, with ammonium transport and assimilation being much faster than for nitrate [21]. An example of the differences between ammonium and nitrate transport potential, and concurrent needs of N assimilation at different levels of N-stress is given in S2 Fig.

The linkage between nutrient transport and assimilation, and ultimately growth, is modulated via the expression of transport capacity for specific nutrient types via end-product (de)repression signals. These events involve both short-term control, for example satiation feedback regulation upon the operation of existing transporter proteins, and longer-term control via synthesis and removal of transporter proteins. This feedback occurs more quickly following ammonium and than nitrate transport because of the rapidity of both ammonium transport and of its assimilation [6,22]. Nitrate may also be accumulated in larger cells further decoupling processes of N-assimilation from transport. Thus, depending on the organism size and nutrient status, the nutrient being tested, experiment sampling, and subsequent data processing methodology, the values of both *G*_*max*_ and *K*_*G*_ may differ significantly from *T*_*max*_ and *K*_*T*_ [6]. Experiments using a given species and nutrient, for example varying the period of N-limitation, may likely give useful information on trends. However, interpretations of inter-species and inter-nutrient differences in *U*_*max*_ and *K*_*U*_, especially when derived by different researchers, carry a high degree of uncertainty.

### Relating transport kinetics to growth kinetics

Estimates of *K*_*T*_ for nutrient transport are very rare, and values for phytoplankton nutrient transporters are rarer still [23], but a value in the range of 0.5–2 μM has been reported [15]. In the following we will assume *K*_*T*_ = 1μM. For comparison, the *K*_*M*_ for enzymes processing biochemical transformations are typically in the mM range [24]. Just as the importance of the numeric value of *V*_*max*_ needs to be placed in the context of the enzyme sample in which it has been measured, so the value of *T*_*max*_ needs to be placed in the context of the cell in which it is located. The value of *T*_*max*_ may be expressed per cell, as a specific transport rate either following N-source uptake using ^15^N, or as a C-specific rate (this is shown in S2 Fig).

Nutrient availability for the cell does not just reflect the bulk water nutrient concentration (*S*_*∞*_), which is readily measured, but it reflects the interactions between processes adding and removing nutrient molecules around the individual cell which affects the substrate concentration (*S*_*0*_) at the transporter protein. Thus *S*_*0*_ is also affected by turbulence, cell size and the cell’s motion [25–27]; collectively these determine the formation of a boundary layer around the cell and thence affect diffusion to the sites of transport. Cell size is a critical determinant in transport kinetics, as it affects the boundary layer thickness and hence the relationship between *S*_*0*_ and *S*_*∞*_. It is thus constructive to express *T*_*max*_ in the context of the surface area of the plasma-membrane in which the transporter proteins reside. If we assume a spherical cell form, with a given equivalent spherical diameter (*ESD*) and an equal distribution of transporter proteins over the membrane surface, we can then report *T*_*max*_ in terms of a transport rate density (*TRD*; Table 1). Thus, for the transport of ammonium-N, units of *TRD* would be g ammonium-N d^-1^ μm^-2^; that is to say that every day across every μm^2^ of cell plasma-membrane area so many g ammonium-N could be transported assuming no satiation feedback. The value of *TRD* is enabled by the activity of many transporter proteins spread over the cell surface area (*SA*), each of which has its own *K*_*T*_ and *k*_*cat*_. *TRD* is thus

$$TRD=\frac{T_{max}}{SA}$$ (2)

and *T*_*max*_ is

$$T_{max}=k_{cat}∙\{transporter\;proteins\;per\;cell\}$$ (3)

The larger the cell, the greater the surface area but there is no reason to necessarily expect the value of *TRD* to differ according to cell size. In the following we ignore changes in cell size associated with nutrient availability (e.g. N-limited cells are typically smaller, while P-limited cells are larger) and environmental conditions (e.g. growth at different temperatures and irradiance [28] affect cell shape and size).

Growth itself is not a simple function of the presence of external nutrient availability (even if estimated more accurately as *S*_*0*_ rather than *S*_*∞*_), but is primarily a function of availability of that nutrient within the cell, and the allied biochemical processes associated with its assimilation into biomass. We thus need to consider transport rates in the context of supply and demand for the cell. Depending on the nutrient status, the value of *T*_*max*_ changes (S2 Fig), and consequentially so does the value of *TRD*. We can now define two important specific values of *TRD*. These are the values of *TRD* needed to enable *G* = *G*_*max*_ (*TRD*_*Gmax*_), and the maximum possible value of *TRD (TRD*_*max*_*)*; the latter defines the value of *TRD* which aligns with the absolute maximum value of *T*_*max*_ (*T*_*absmax*_) which is usually expressed by a cell at an intermediate level of nutrient stress (S2 Fig). By analogy with the plots shown in S2 Fig, we can also consider the excess in transport potential that develops during nutrient stress as the ratio of *TRD*_*max*_: *TRD*_*Gmax*_ (δ_*TRD*_; Table 1).

At saturating concentrations of nutrient and plausible maximum growth rates we can assume that diffusion is not limiting the supply of substrate to the transporter proteins (S_0_ ≈S_∞_), and hence we can estimate the value of *T*_*max*_ (as per S2 Fig) and hence *TRD*. From experimental work for ammonium and nitrate transport into the coccolithophorid *Emiliania huxleyi*, raphidophyte *Heterosigma carterae* and the diatom *Thalassiosira weissflogii* we compiled the data shown in Table 2. These values exploit relationships between cell biovolume measured using an Elzone (Coulter counter–like) instrument, and C-biomass derived from elemental analysis. In Table 3, comparative values for *TRD* are presented, calculated using the allometric relationships of cell size to C-content taken from the literature [9]. While there are significant differences between the C-, and thus the N-content of the cells computed according to these different methods, from these estimates we obtain a feel for a likely maximum value of *TRD*_*max*_. For a given computational choice (Table 2 or Table 3) the value of *TRD*_*max*_ is not so different between organisms of markedly different taxonomy, size and maximum growth rate potential. These values suggest a decreasing scope for excess in transport potential *δ*_*TRD*_ (i.e., *TRD*_*max*_: *TRD*_*Gmax*_) with increasing size, which may be expected, given the associated changes in surface area to volume (*SA*:*Vol*) ratio.

**Table 2:** Allometric, stoichiometric and ammonium transport characteristics for 3 phytoplankton species. **More detailed explanations of the variables are given in Table 1.** The data have been compiled from [29–34] for the coccolithophorid *Emiliania huxleyi*, raphidophyte *Heterosigma carterae* and the diatom *Thalassiosira weissflogii*.

Variable	Unit	*Emiliania*	*Heterosigma*	*Thalassiosira*
*ESD*	μm	4.5	11.5	13.9
*Volume*	μm^3^ cell^-1^	47.7	800.0	1400.0
*SA*	μm^2^ cell^-1^	63.62	416.75	605.21
*cellular carbon density*	gC (L cell)^-1^	258	280	330
*C*_*cell*_	pgC cell^-1^	12.31	224.0	462.0
*NC*_*max*_	gN (gC)^-1^	0.15	0.18	0.18
*G*_*max*_	d^-1^	1.4	0.44	1.4
*NCT*_*Gmax*_^*1*^	gN (gC)^-1^ d^-1^	0.21	0.0792	0.252
*NCT*_*max*_^*2*^	gN (gC)^-1^ d^-1^	1	0.28	0.5
*TNcell*_*Gmax*_^*3*^	pgN cell^-1^ d^-1^	2.585	17.741	116.424
*TNcell*_*max*_^*4*^	pgN cell^-1^ d^-1^	12.31	62.72	231.0
*TRD*_*Gmax*_	pgN μm^-2^ d^-1^	0.0406	0.0425	0.1924
*TRD*_*max*_	pgN μm^-2^ d^-1^	0.1935	0.1505	0.3817
*δ*_*TRD*_	*TRD*_*max*_: *TRD*_*Gmax*_	4.76	3.54	1.98

^*1*^ *NCT*_*Gmax*_*−*N transport rate expressed per cell-C required to support *G* = *G*_*max*_

^2^ *NCT*_*max*_−maximum possible N transport rate expressed per cell-C

^3^ *TNcell*_*Gmax*_—N transport rate expressed per cell required to support μ = μ_*max*_

^4^ *TNcell*_*max*_*—*maximum possible N transport rate expressed per cell.

**Table 3:** Alternatives to Table 2 computed using an allometric scaling function. More detailed explanations of the variables are given in Table 1 and legend of Table 2.

Parameter^*1*^	Unit	*Emiliania*	*Heterosigma*	*Thalassiosira*
*a*		0.261	0.261	0.288
*b*		0.86	0.86	0.811
*cellular carbon density*	gC (L cell)^-1^	151.93	102.38	73.24
*C*_*cell*_	pgC cell^-1^	7.25	8.19	102.54
*TNcell*_*Gmax*_	pgN cell^-1^ d^-1^	1.52	6.49	25.84
*TNcell*_*max*_	pgN cell^-1^ d^-1^	7.25	22.93	51.27
*TRD*_*Gmax*_	pgN μm^-2^ d^-1^	0.0239	0.0156	0.0427
*TRD*_*max*_	pgN μm^-2^ d^-1^	0.1139	0.0550	0.0847

^*1*^ Relationships of the form *C*_*cell*_ = a*(4/3*π*(*ESD*/2)^3^)^b^, where the values of *a* and *b* (as tabulated here) come from [9]. All other abbreviations are explained in Table 2.

An analysis of the data compiled by [11], which reports experimentally derived nutrient uptake maxima, and assuming *U*_*max*_ = *T*_*max*_, yields average *TDR*_*max*_ that are broadly in line with those in Tables 2 and 3. Those data yield *TRD* values (as pg nutrient μm^-2^ d^-1^) of 0.075 (+/- SE 0.041), 0.115 (+/- SE 0.0137) and 0.172 (+/- SE 0.069) for ammonium-N, nitrate-N and phosphate-P uptakes, respectively, with no statistical relationship with *ESD*. It is noteworthy that the *TRD* values for ammonium estimated from the data compiled by [11] are half those for nitrate; ammonium *T*_*max*_ and thus *TRD* is expected to be much greater than the values for nitrate transport [21,34], which could indicate confounding estimation of kinetic parameters by different researchers, as explained earlier.

Ultimately the balance of supply and demand is reflected in how close an organism comes to attaining its maximum growth rate, *G*_*max*_. It is this maximum rate of growth, and the form of the functional curve relating nutrient concentration to the achieved growth rate (*G*) that help define competitive advantage, and certainly do so in simple mathematical models. However, while the performance of each transporter protein may be expected to conform to the RHt2 equation of Michaelis-Menten kinetics, diffusion limitation is expected to decrease potential transport at lower nutrient concentrations [35,4,36], and the satiation feedback is expected to suppress transport rates at higher concentration (S2 Fig). In short, there are various reasons to expect that a RHt2 response curve (as used in simple models) will not well describe the true functional response curve between the bulk nutrient concentration (*S*_*∞*_) and *G*. Indeed, we should likely not expect such a RHt2 relationship even between *S*_*0*_ and *G* (S1 Fig).

Let us now consider the situation that aligns with a growth rate at half the maximum value (*G*_*0*.*5*_). At this rate, the residual steady-state nutrient concentration in the bulk medium (*S*_∞_) would equate to the half saturation value for growth, which defines *K*_*G*_. The value of *T*_*max*_ in cells growing at the N-status equal to *G*_*0*.*5*_ is much higher than the value of *T*_*max*_ in cells growing at *G*_*max*_ (S2 Fig; e.g. [21]). In addition, the amount of N required to support growth at *G*_*0*.*5*_ is less than that required to support *G* = *G*_*max*_. If, for example, we consider *G*_*max*_ to be associated with a maximum cellular N:C (g:g) of 0.2, and *G* = 0 with a minimum N:C of 0.05, then a cellular N:C aligning with *G*_*0*.*5*_ would be expected to be ca. 0.125 gN gC^-1^ (S2 Fig). In such a situation, the potential excess (*δ*_*TRD*_) in transport capacity, of *T*_*max*_, at *G*_*0*.*5*_ could be ca. 20 fold the nutrient transport rate required at *G*_*max*_. It is thus readily apparent that cells with different stoichiometries will exhibit different growth kinetics with respect to nutrient concentration, all else being the same.

There is one other important part of the jigsaw, and that concerns the relationships between cell size, the cellular carbon density as affected by vacuolation, and cell shape. For simplicity we assume a spherical cell, which then sets surface area (*SA*) as a simple geometric function of cell size (*ESD*). Vacuolation in protists, and especially in diatoms, increases markedly with *ESD* [9,37], and hence the demands for nutrient transport across each μm^2^ of cell surface does not simply relate to cell size.

Having described the physiological framework, and considered the experimental data, we now proceed to extend the analysis according to allometric constraints across a range of sizes, organism types and motilities. The questions that we consider are:

1. How may allometry, stoichiometry and changing cellular carbon density (vacuolation) affect *K*_*G*_?
2. How may motility or sedimentation rates affect *K*_*G*_?
3. How may the maximum growth rate (*G*_*max*_) affect *K*_*G*_?

The emphasis here is on factors that impact upon *K*_*G*_, namely *S*_*∞*_ that support *G*_*0*.*5*_. This value of *K*_*G*_ can be seen to be an emergent property of *TRD*, *K*_*T*_, cell size, *G*_*max*_, cell motility, cell vacuolation and cellular elemental stoichiometry. To our knowledge, no previous study has considered the interconnected nature of all these facets. Collectively these also embrace the core features considered in classic trait trade-off studies.

## Results

**Fig 1:**
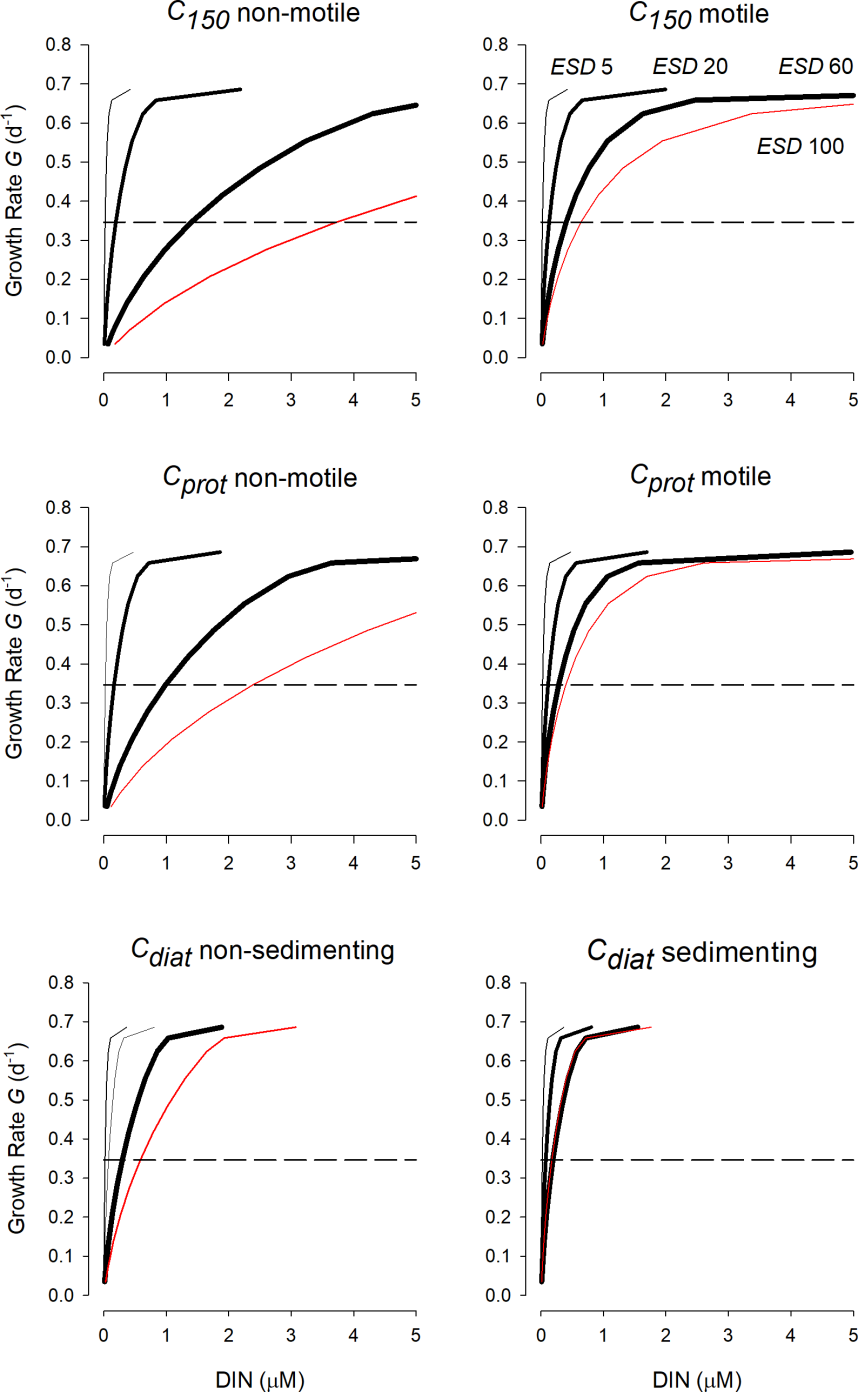
Plots of the potential growth rate for cells of different size against bulk nutrient concentration. In all instances the maximum growth rate is set at *G*_*max*_ = 0.693 d^-1^ (one doubling per day, assuming a constant rate of N-transport over the day). Organism configurations shown represent cells with a cellular carbon density which is fixed (*C*_*150*_), which accords with a generic protist phytoplankton (*C*_*prot*_) or with a diatom (*C*_*diat*_). More details are given in Table 1. *TRD*_*max*_ = 0.4 pgN μm^-2^ d^-1^. The dashed horizontal line indicates *G = G*_*max*_/2 = *G*_0.5_; the corresponding value of DIN is *K*_*G*_.

Fig 1 shows the potential growth rate at given external bulk nutrient concentrations (*S*_*∞*_) in terms of dissolved inorganic-N, DIN, for different cell types and configurations, all with the same fixed maximum growth rate of *G*_*max*_ = 0.693 d^-1^. These plots clearly show the competitive advantage for nutrient transport of being small, and of motion achieved through either swimming (flagellated phototrophic protist) or sedimentation (diatom). Thus the value of *S*_*∞*_ supporting *G*_*0*.*5*_ (*G* = 0.693/2 = 0.346 d^-1^), which is the value of *K*_*G*_, decreases with cell size and with motion. At cell *ESD* below 5μm, at this growth rate, nutrient concentrations at the cell surface are similar to those in the bulk water. The cellular carbon density also has an important impact on the growth-nutrient kinetics; increasing vacuolation with size (for a given C:N stoichiometric configuration) decreases the requirement for N transport. It is thus apparent that diatoms can compensate significantly for increasing cell size through being more vacuolate and hence having *de facto* a lower than expected *SA*: cell-N ratio compared to a typical protist phytoplankton. While altering the value of *K*_*T*_ (assumed by default as 1μM) changes *K*_*G*_, the relationship is not *pro rata*; thus halving *K*_*T*_ decreases *K*_*G*_ to ca. 75%, and doubling *K*_*T*_ increases *K*_*G*_ to ca. 150%.

**Fig 2:**
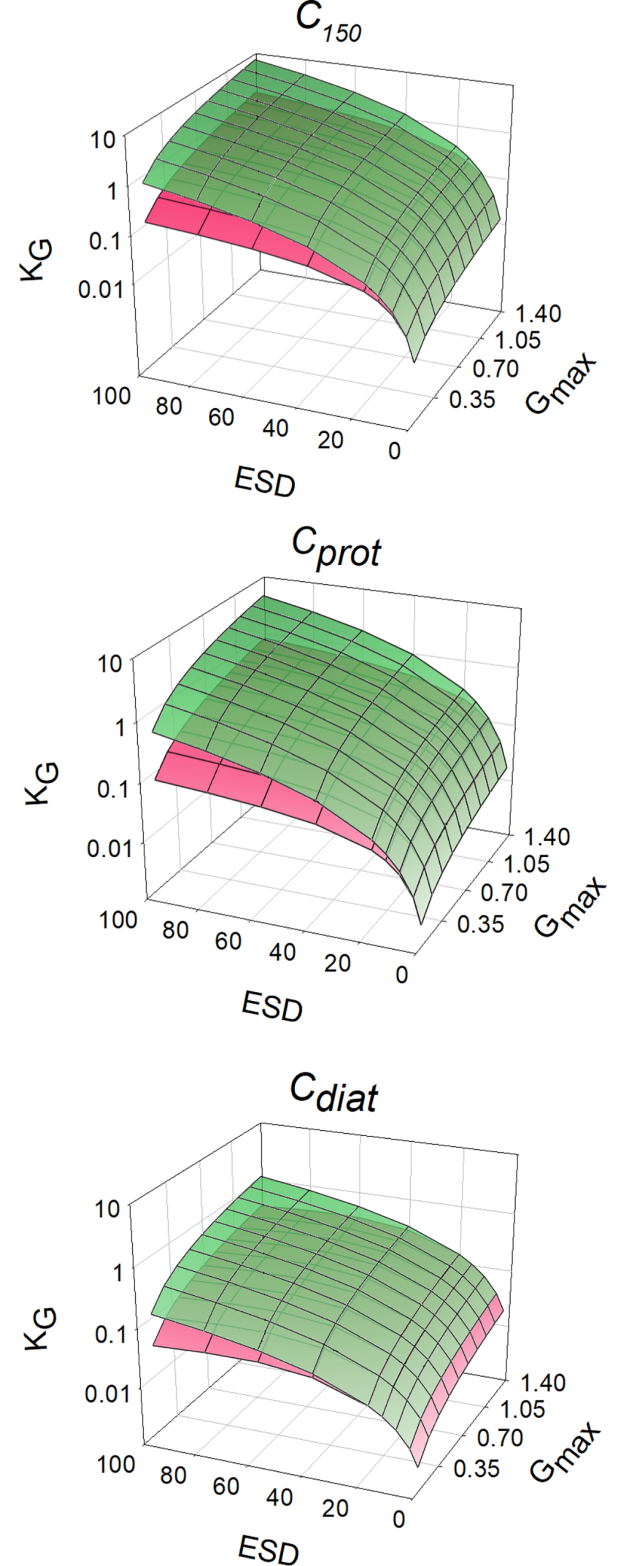
Relationship between cell size and the resultant value of *K*_*G*_. Organism configurations are shown representing a cellular carbon density that is fixed (C_150_), and accords with a generic protist phytoplankton (*C*_*prot*_), or with a diatom (*C*_*diat*_). More details are given in Table 1. The green layer is for non-motile (non-swimming or non-sedimenting) cells; the pink layer is for motile (non-diatom protist; Eq 12 in Methods), or sedimenting (diatoms; Eq 13 in Methods) cells. Note that the *K*_*G*_ scale is logarithmic.

**Fig 3:**
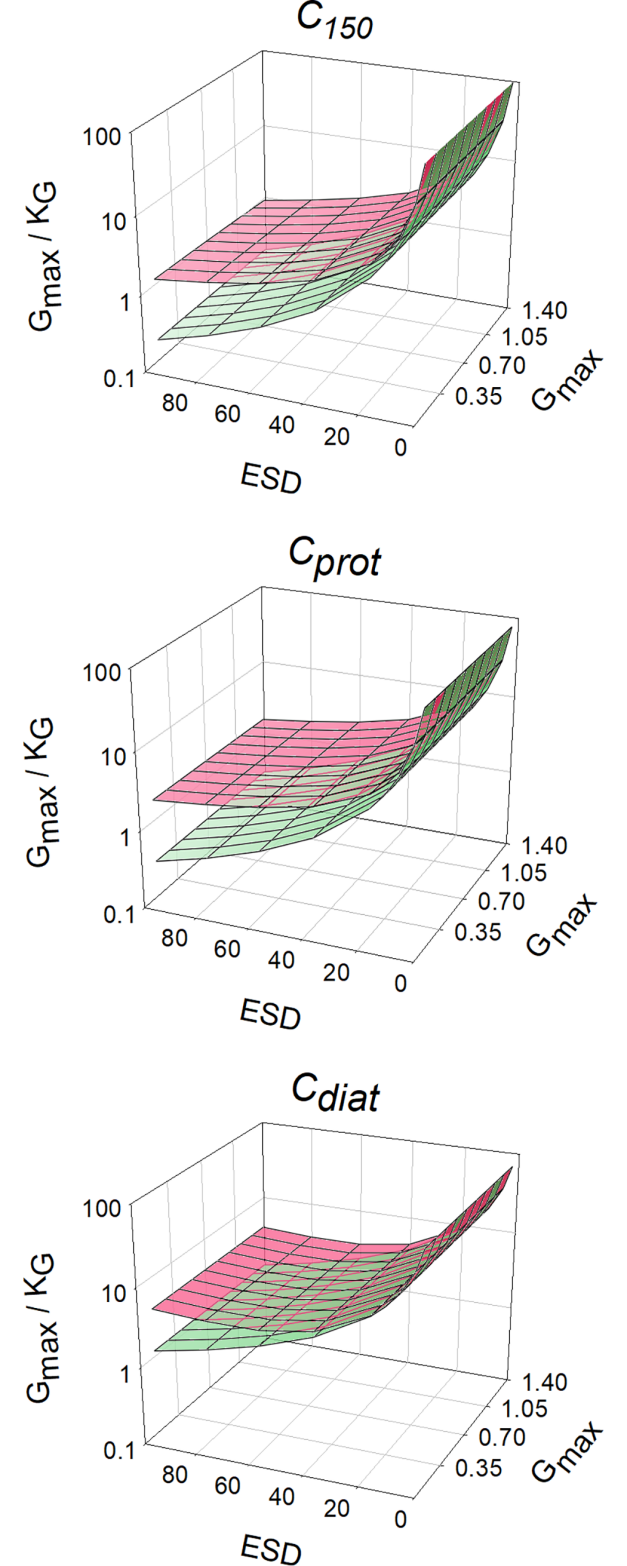
ESD vs μ_max_ and their resultant values of *G*_*max*_/*K*_*G*_. Developed from Fig 2, this plot shows organism configurations representing a cellular carbon density that is fixed (*C*_*150*_), accords with a generic protist phytoplankton (*C*_*prot*_), or with a diatom (*C*_*diat*_). More details are given in Table 1 and in the legend for Fig 2.

**Fig 4:**
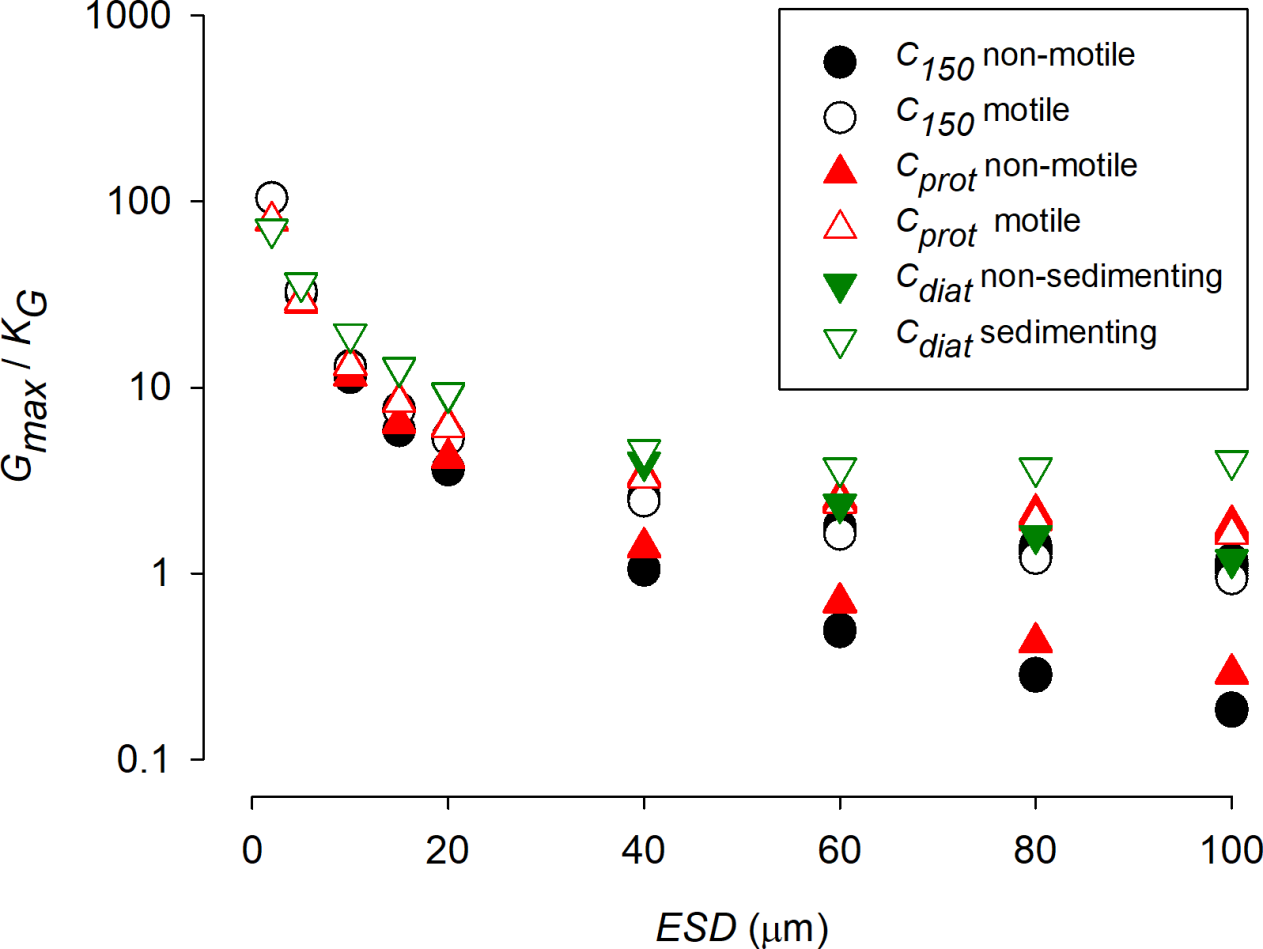
Developed from Fig 3, this plot shows *ESD* vs *G*_*max*_/*K*_*G*_. Table 4 shows the power-regression for best fit through these data. See legend for Fig 2 for further information.

In Fig 2, values of *K*_*G*_ obtained with different cell configurations growing with different maximum growth rates are plotted, showing that smaller cells can attain a higher *G*_*max*_ relative to *K*_*G*_; their value of *G*_*max*_/*K*_*G*_ is higher. Fig 3 also shows the potential for cell motion and/or cellular carbon density to compensate for the negative impact of increasing *ESD*. For a given cell configuration, however, the value of *G*_*max*_/*K*_*G*_ is invariant with changing *G*_*max*_ (Fig 3). The negative relationship between *G*_*max*_/*K*_*G*_ and *ESD* varies strongly between cell configurations, and becomes more variant between configurations at larger *ESD* (Fig 4). The power slopes between *G*_*max*_/*K*_*G*_ and *ESD* are given in Table 4; assuming a cellular carbon density that is fixed (*C*_*150*_), or accords with a generic protist phytoplankton (*C*_*prot*_) or with a diatom (*C*_*diat*_). More details regarding the organism’s configuration are given in Table 1. The slope exceeds -1.5, but motility (through swimming or sedimentation) and increasing vacuolation with ESD mitigate the slope to closer or less than -1.

**Table 4:** Power-regression for (*G*_*max*_/*K*_*G*_ = a * *ESD*^b^) best fit through the data shown in Fig 4. Further explanations regarding the organism configuration and motility scaling are provided in Table 1.

Organism configuration	Motility	a	b	R^2^
*C*_*150*_	non-motile	428.57	-1.645	0.9947
	motile	206.7	-1.173	0.9949
*C*_*prot*_	non-motile	284.7	-1.459	0.9934
	motile	135.06	-0.979	0.9918
*C*_*diat*_	non-sedimenting	196.12	-1.073	0.9878
	sedimenting	119.47	-0.808	0.9732

**Fig 5:**
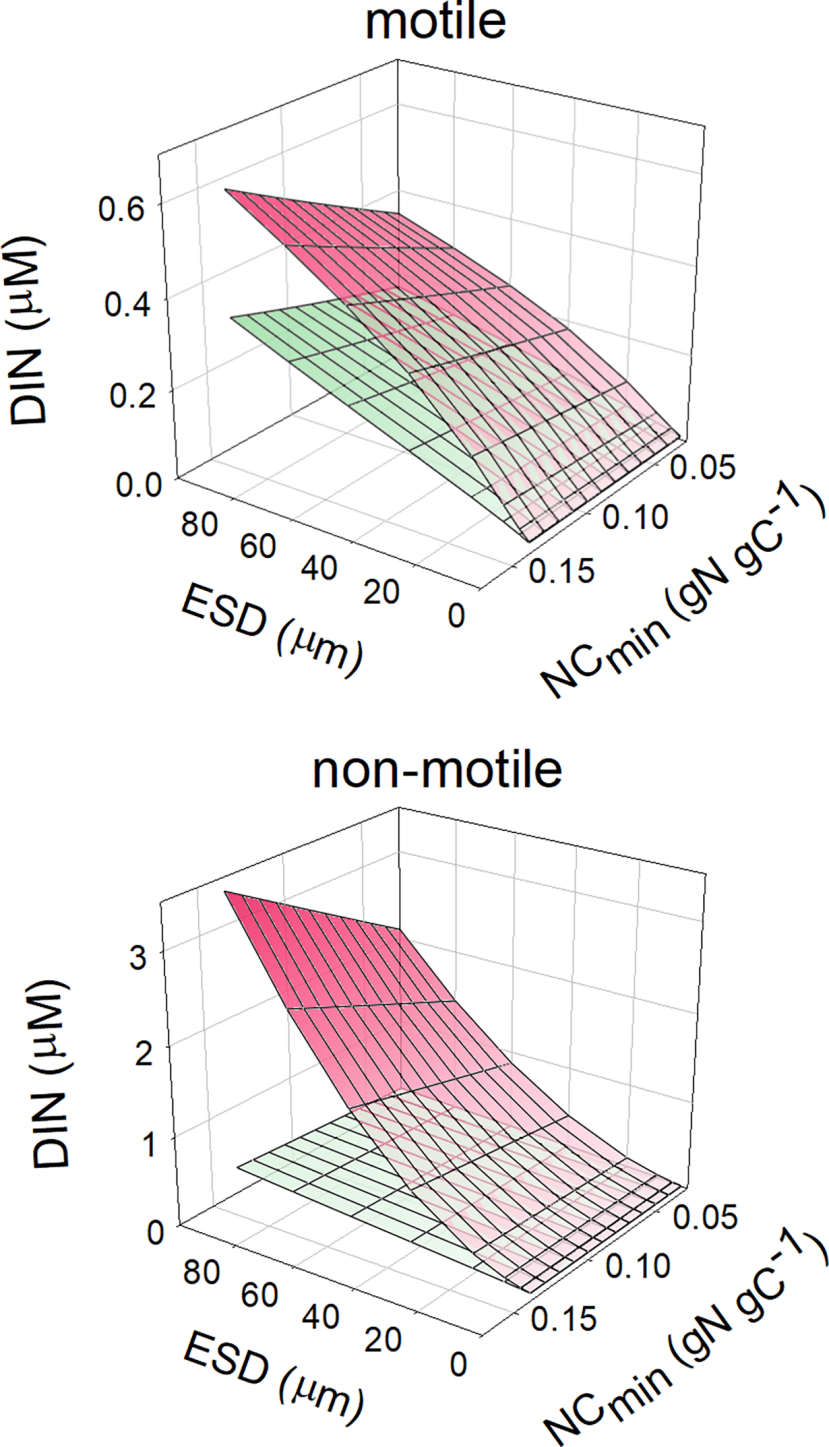
Relationship between the minimum N:C quota, cell size, and the DIN concentration required to support *G*_*0*.*5*_. *G*_*max*_ = 0.693 d^-1^ for motile and non-motile protists alike. The green layer is for DIN at the cell surface (*S*_*0*_), and is the same in both plots; the red layers are for DIN in the bulk medium (*S*_*∞*_), and thus is the value of *K*_*G*_. In all instances, *NC*_*max*_ = 0.18.

To consider the implications of variable elemental stoichiometry, Fig 5 presents the relationship between cell size and the minimum N:C quota (*NC*_*min*_) and the nutrient concentration that half saturates transport of dissolved inorganic-N for protists (non-diatoms) that are motile or non-motile. This assumes a fixed maximum growth rate and fixed maximum N:C quota. These plots demonstrate a linear increase in *K*_*G*_ as the difference between *NC*_*max*_ and *NC*_*min*_ decreases; cells with a more restricted N:C quota need more N and thence are disadvantaged if DIN acquisition is the sole limiting factor.

**Fig 6:**
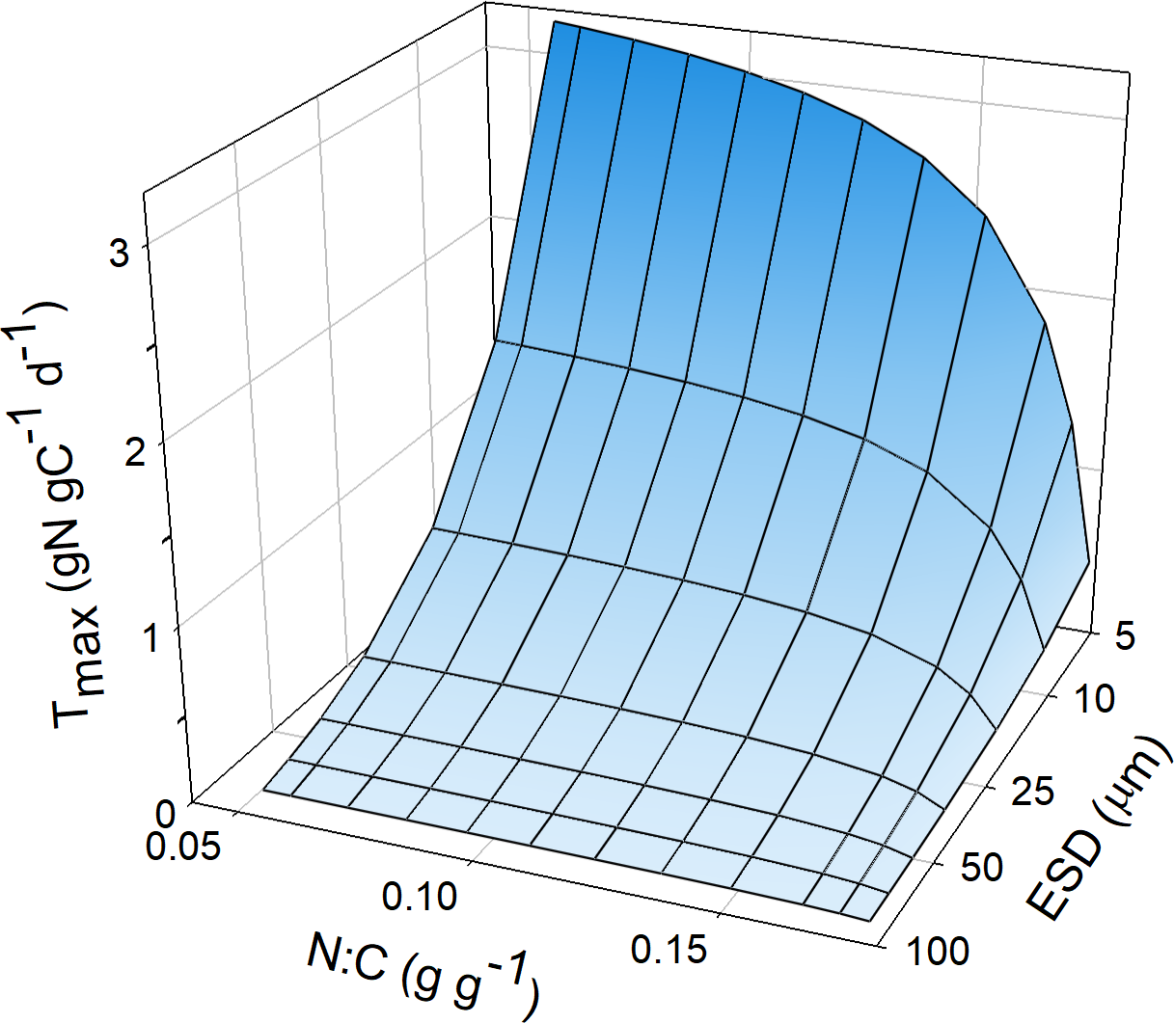
Relationship between N-status (N:C) and *T*_*max*_ for cells of different size. Calculations were undertaken using Eqs 5, 6 and 7. Here *G*_*max*_ = 0.693 d^-1^, *KT*_*con*_ = 0.1, maximum and minimum N:C at 0.18 and 0.05, respectively; the cellular carbon density was assumed to be fixed at 150gC (cell L)^-1^ (i.e., *C*_*150*_); the maximum transporter rate density was set at *TRD*_*max*_ = 0.4 pgN μm^-2^ d^-1^.

S3 and S4 Figs show how N-specific transport (which aligns with growth rate) varies with nutrient concentration for cell configurations *C*_*prot*_ and *C*_*diat*_, considering different maximum growth rate potentials, *ESD*, and different relationships between N-status and *T*_*max*_. These plots show how the difference between bulk water and cell surface nutrient concentrations (*S*_*∞*_ vs *S*_*0*_) for a given transport rate increases with *ESD* and with maximum growth rate. Also apparent is that, for a given *K*_*T*_ (all these plots assuming the same value of 1 μM) the relationship between N-status and *T*_*max*_ has a very significant effect on the kinetics (as expected from S1 Fig). To consider whether these kinetics could be adequately described through application of a simple RHt2 response curve (as per Eq 1), such a curve form was fitted to the model output using an iterative approach (as supported by SigmaPlot 12.5); the fit assumed either a free maximum rate, or a maximum rate that is fixed equal to the value of *G*_*max*_. Especially notable, where *T*_*max*_ increases with deteriorating N-status (Fig 6), is that the form of the response curve appears steeper and/or plateaus more abruptly than for a RHt2 curve (S3–S6 Figs). Nonetheless, the R^2^ values for all of these fits exceed 0.98. The RHt2 plots typically overestimate transport at nutrient concentrations aligning with the value of *K*_*G*_ and could significantly over-estimate (free-fitting maximum; “RHt2” plots in S3–S6 Figs) or under-estimate (plateau fixed equal to *G*_*max*_; “RHt2 fGmax” plots in S3–S6 Figs) transport at higher nutrient abundance.

**Fig 7:**
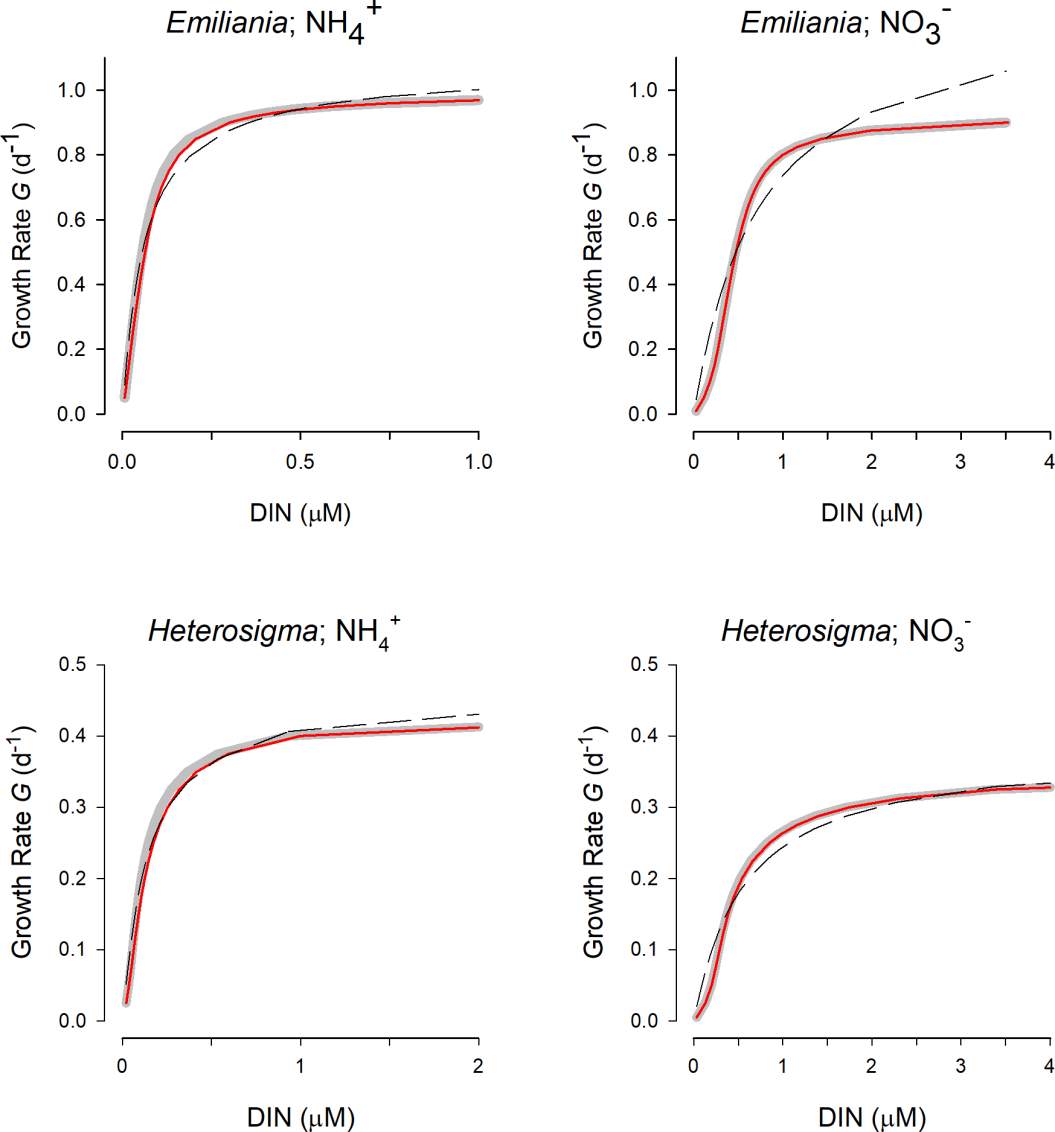
Relationship between ammonium or nitrate concentration and growth rate for *Emiliania* or *Heterosigma*. The response curves relating *T*_*max*_ to nutrient status, and nutrient status to growth rate were as in S2 Fig. The grey curve indicates the relationship at the plasma-membrane surface; this relationship would also apply if diffusion limitation was zero (i.e., ignored). The solid red curve is the relationship with the bulk nutrient concentration. The dashed black curve is the rectangular hyperbolic type 2 (RHt2) fit through the data describing the red curves with unconstrained fitted values of *T*_*max*_ and *K*_*G*_. Note the different x-axis ranges.

Rather than using simple hypothetical relationships between N-status and *T*_*max*_ (S3 Fig and S6 Fig), in Fig 7 experimentally derived response curves (from S2 Fig) were deployed. Again, the importance of the form of the relationship between N-status and *T*_*max*_ is clear; especially for the nitrate curves, the deterioration in transport capacity at low N-status (low N:C in S2 Fig) results in the cell-surface nutrient values being closer to the bulk water values than may otherwise have been expected. Fig 7 also shows how RHt2 curves that give statistically acceptable fits also give differences in projected transport rates for a given nutrient concentration that could be significant in simulations. This is especially so for nitrate-supported growth.

## Discussion

The relationship between resource abundance and growth rate (hereafter, the “RG-relationship”) is widely considered as a key factor affecting competitive advantage, as represented as a core theme in ecological research [38]. Not only does the relationship affect bottom-up regulations in a direct fashion but it affects organism health and nutritional status, and thus affects ecological stoichiometry [39,40]. The analysis presented here indicates very significant scope for variation in the RG-relationship for phytoplankton, linked to cell elementary stoichiometry, cell size, maximum growth rate potential, motility or sedimentation, cellular carbon density (vacuolation) and the enhancement of transport potential with nutrient stress. The situation is complicated further given that we now recognise that many phytoplankton are mixotrophic, not only using inorganic nutrients but also being capable of using organic compounds and contributing to their resource needs through predation [41]. Nonetheless, the RG-relationship has been, and will continue to be, deserving of attention as it impacts on so many facets of competition within plankton communities [3] and in general ecology.

### “Affinity” and competitive advantage in nutrient transport

We may consider that transporter proteins are specialist enzymes. There is an established literature exploring the competitive advantages, and evolution, of enzymes of different *k*_*cat*_ and *K*_*M*_. Pettersson (1989) [42] considers the evolution of the value of *k*_*cat*_/*K*_*M*_ noting that, beyond the initial phase that sees the expected increase in *k*_*cat*_ and decrease in *K*_*M*_, enzyme evolution displays a linked increase in both *k*_*cat*_ and *K*_*M*_; the value of *k*_*cat*_/*K*_*M*_ approximates the diffusion control limit at the level of the enzyme molecule. Several studies [43–45] discuss the usefulness of this so-called “specificity constant” (*k*_*cat*_/*K*_*M*_) pointing out various problems both with the usefulness of the value itself, and with its some-time alternative title as a value of “catalytic efficiency”.

Interpretations of transporter kinetic parameters, operating at the site of individual transporter proteins, would be similarly implicated in such considerations. Just as trying to piece together whole organism biochemical evolution through reference to *k*_*cat*_/*K*_*M*_ for all the constitutive enzymes in an organism is fraught with problems [42], so too are considerations of transport kinetics for different substrates into different species. However, it is noteworthy that our analysis indicates that, for a given cell configuration (size, motility, value of *C*_*cell*_, stoichiometry; Fig 6 and Fig 7), the value of *G*_*max*_/*K*_*G*_ is constant, as is *k*_*cat*_/*K*_*M*_ expected to be constant in an evolutionary mature enzyme.

The phytoplankton literature has hitherto explored the relative competitive value of organisms under nutrient limitation through reference to (in our terminology–see Table 1) to *U*_*max*_/*K*_*U*_. This value of *U*_*max*_/*K*_*U*_ has been termed “affinity” in parts of this literature [10,46]. Such usage of “affinity” conflicts with traditional parlance for enzyme affinity, which defines affinity by just the half saturation constant *K*_*M*_. The form and interpretation of *U*_*max*_/*K*_*U*_ is also different to that for *k*_*cat*_/*K*_*M*_ for enzymes; while *K*_*U*_ may approximate to *K*_*M*_, *U*_*max*_ is *de facto* a function of the product of transporter *k*_*cat*_ and the number of transporter proteins. The number of transporter proteins varies with cell size, nutrient status and likely also with *G*_*max*_. In addition, there is the practical challenge of measuring *U*_*max*_, being as it is a function of *T*_*max*_ (the rate of transport at the start of the experimental incubation, at t_0_; [6]) and incubation conditions during the assay. In consequence, the values of *U*_*max*_ and *K*_*U*_, and thence of their ratio, are subject to various confounding issues. The value of *U*_*max*_/*K*_*U*_ could, under ideal conditions of measurement, perhaps be equated to *T*_*max*_/*K*_*T*_; however, there is still the question as to the impact of nutrient status upon *T*_*max*_ (S2 Fig), and the complication that *K*_*T*_ is the substrate value at the transporter protein (*S*_*0*_) while *K*_*U*_ is the value of the substrate concentration in the bulk medium (*S*_*∞*_).

The underlying explanations and potential trade-offs in expression of the uptake affinity defined as *U*_*max*_/*K*_*U*_ has been argued to lack a mechanistic basis, hence leading to a potential misrepresentation of primary production in modelling approaches [3,47,5]. Our results indicate why a search for such a mechanistic basis has proven so difficult; there are too many confounding factors. An alternative approach considers nutrient uptake as a function of cell traits and actual nutrient availability in a turbulent environment [4,48,49]. The non-linear formulation describes so-called affinity as a function of cell size, density of uptakes sites at the cell surface (i.e. transporter proteins) and turbulence [5]. This diffusion- limited nutrient uptake results in a linear scaling of affinity with the cell diameter or radius (*r*). While some experimental results are consistent with this scaling [50], the general picture drawn by laboratory experiments over a wide range of sizes of taxa indicate a scaling closer to the square of cell radius [10,51] that is with the cells surface area, a trend that becomes more pronounced with decreasing cell size. Theoretical arguments have suggested that this mismatch might stem from the fact that cells are not “perfect sinks”, hence are not able to absorb all nutrients at the cells surface immediately as assumed by diffusion limited nutrient uptake [20], which is likely once satiation feedback develops. According to these considerations, while smaller cells are favoured by a larger surface to volume ratio, they also require a higher transporter density to achieve maximum affinity and would thus have higher relative investment costs [20]. However, *T*_*max*_ increases during at least the initial phase of nutrient-limitation (S2 Fig), which demonstrates an increased synthesis cost for transporters in such nutrient-limited cells; this suggests that the investment cost in transporters is not significant. There are clearly challenges with all the above analyses, centring upon what exactly *U*_*max*_ and *K*_*U*_ index as curve-fitting parameters for RHt2 curves fitted through imperfect (and only partially understood) experimentally-derived data.

With suitable methods, estimates of *U*_*max*_ will approach *T*_*max*_, and estimates of *K*_*U*_ will approach *K*_*T*_ [6]. The numeric disparity between these variables depends on the nutrient status of the cell, the size of the cell (and thus how close *S*_*0*_ is to *S*_*∞*_), the form in which the nutrient is available, and the capacity of the cell to accumulate unaltered that particular nutrient prior to the development of satiation feedback. In consequence, greater challenges could be expected when measuring the kinetics of ammonium transport, which is assimilated very rapidly [8] and not accumulated. The ability of the diatom *Phaeodatylum* to take up the un-metabolisable ammonium analogue methlyamine is many orders of magnitude higher than for any other N-source [21]. This likely reflects the fact that methylamine entering via the ammonium transporter is not subject to the usual very rapid accumulation of the ammonium-transport-repressor signalling amino acid glutamine [8]. Lesser problems can be expected when measuring nitrate transport into a large vacuolated diatom that may accumulate nitrate [52], in comparison to transport into a nanoflagellate that lacks such vacuoles. It may therefore likely be no coincidence that the (few) data for kinetics for ammonium transport collated by Edwards *et al*. (2015) [11] appear so competitively poor in comparison with those for nitrate when the converse might have been expected. Similarly we expect fewer challenges when measuring phosphate transport into a cell type that accumulates polyphosphate.

**Fig 8:**
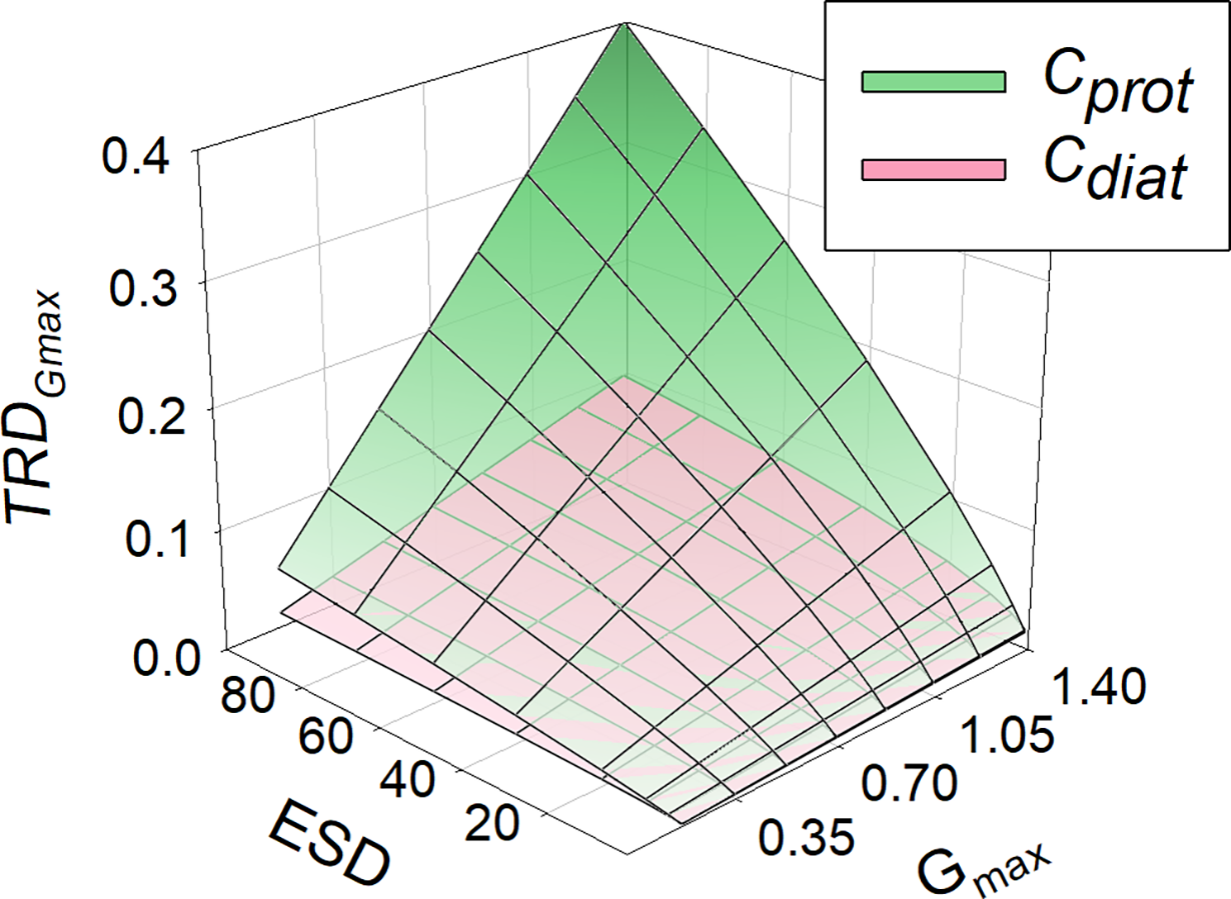
Relationship between *G*_*max*_, cell size, and *TRD*_*Gmax*_. Maximum N:C (at *G* = *G*_*max*_) was assumed as 0.18 gN gC^-1^.The required value for *TRD*_*Gmax*_ in C_diat_ is less than that for C_prot_ because diatoms, being more vacuolated with a lower gC (cell L)^-1^, have a decreased demand for N across a given area of cell plasma-membrane. The absolute maximum value of TRD (*TRD*_*max*_) is expected to be ca. 0.4 pgN μm^-2^ d^-1^; large fast-growing protists approach the limit of *TRD*_*Gmax*_ = *TRD*_*max*_.

Nutrient “affinity” [10,46], which has been described in our terminology as *U*_*max*_/*K*_*U*_, has typically not been related to the C:N:P stoichiometry of the cell nor to the cellular carbon density both of which will affect the numeric value of this index. Together, these additional data would provide links between nutrient-status and *T*_*max*_ and to the level of vacuolation affecting resource demand to be satisfied by transport over the cell surface. Collectively, stoichiometry (Fig 5) and cellular carbon density (Fig 1) affect the cell’s demand for the nutrient, which is a critical factor affecting the relative importance of any index of nutrient affinity. There is, however, scope for *T*_*max*_ to vary allometrically on account of the packing of transporter proteins within the plasma membrane (Fig 6); which is consistent with the suggested explanation of the discrepancy between theoretical scaling and observed values of *U*_*max*_/*K*_*U*_ [20]. Further, and of greater significance for large non-diatoms protists than for diatoms, there is scope for the maximum growth rate to be limited by *TRD* attaining *TRD*_*max*_ (Fig 8). That is, if *TRD*_*max*_ = *TRD*_*Gmax*_ there is no scope to further enhance transport during nutrient stress. This is important because the value of *K*_*G*_ is a function of the potential transport over the required capacity in transport (S1 Fig), as the ratio *T*_*max*_: *T*_*Gmax*_. This means that larger cells, and faster growing cells of a given configuration (cell type and motility), are expected to have a higher *K*_*G*_.

There are also additional features of ecophysiology that affect the medium term dynamics of nutrient transport. There is for example a difference in the handling of ammonium versus nitrate, that sees the uptake and assimilation of ammonium more constrained to just the light phase. Thus ammonium transport rates during light may have to be double those expected looking at the day-average value, while nitrate assimilation is more likely split over the whole day [30]. In these contexts, it is interesting to note the relationships between *ESD* and *G*_*max*_ for different cell types [53], and that the typical value of *G*_*max*_ in phytoplankton equates to a division per day (*G*_*max*_ = 0.693d^-1^), aligning with RuBisCo activity [19]. It is not just nutrient acquisition at nutrient-limiting concentrations that may be limiting growth rate potential; maximum transport at non-limiting concentrations may also be a factor (Fig 8). While for nitrate transport, there may be the potential for the expression of high-rate transporters, endowing the cell with a biphasic kinetic capability [18,54,55], this may be less likely for ammonium transport. Ammonium is highly toxic at high internal concentrations and its transport appears, unsurprisingly, tightly regulated. Ammonium is also normally present at low (often at vanishingly low) concentrations in natural waters, as the product of N-regeneration in ecosystems with low inorganic N concentrations. If for a given cell, the ammonium transporter exists only as a high affinity system, which is incapable of supporting growth at the highest rates because of limitations in *TRD* for ammonium, then high growth rates in large protists may only be possible when augmented by nitrate transport. This would place an interesting new spin on our understanding of ammonium-nitrate interactions, with implications for modelling biogeochemical and ecological events.

The results of our analysis show how features relating to the regulation of the synthesis and kinetics of transporter proteins, as well as to stoichiometric and allometric features of the cell, all play a part in the story. Arguably, the competitive advantage of an organism would be best indexed by the value of *G*_*max*_/*K*_*G*_ as this integrates over all aspects of the organism’s nutrient physiology. We thus emphasise factors affecting *K*_*G*_. In the following we assume for the most part that all else remains constant (i.e. *K*_*T*_, *TRD*_*max*_, *NC*_*max*_ and *NC*_*min*_ are constant) and consider the impacts of each of these factors upon the system.

### Allometry, stoichiometric, and cellular carbon density effects on *K*_*G*_

If the cellular carbon density is constant across cell sizes, then there is a clear and powerful impact of cell size on *K*_*G*_ (Figs 4–7). Smaller cells are much better equipped than are larger cells in this regard; this is because the *SA*:*Vol* ratio directly translates to a *SA*:N-demand ratio, as well as to lower diffusion limitations in smaller cells [56]. However, in reality there is an important allometric relationship between cellular carbon density and cell size [9] such that larger cells have a lower cellular carbon density. For diatoms in particular, which are increasingly vacuolate at large size [37], this greatly decreases the needs for nutrient transport across a given area of plasma-membrane. According to the calculations presented here, large diatoms with high sedimentation rates appear potentially to be much better adapted to make use of low nutrient concentrations than one may expect if one was to assume a fixed cellular carbon density (i.e., *C*_*diat*_ vs *C*_*150*_) (Fig 4).

The consequences of this decrease in cellular carbon density with cell size is actually secondary to the decrease in N-cell density; the above mentioned mitigation of cell size on *K*_*G*_ in consequence of the lower N-cell density thus assumes that cell stoichiometry is the same. From the effects of altering the range of cell stoichiometry, shown in Fig 5, we conclude that cell stoichiometry and the form of the relationship between stoichiometry and growth rate (the quota relationship–see [57]) are also important factors to consider when reviewing calculations of *K*_*G*_. That is to say, if larger cells had a high *NC*_*min*_, such that the value of N:C at *G*_*0*.*5*_ was elevated, then the mitigation afforded through being more vacuolated would be eroded. Conversely, if smaller cells were relatively N-rich, then the advantage of being small would be eroded. For example cyanobacteria are typically relatively N-rich [58] and would therefore not be so competitive as may at first appear.

The physiology of nutrient acquisition and stoichiometry has the potential to override, or at least partially compensate for, limitations at transport [59]. Models considering detailed explorations of nutrient uptake kinetics thus need also to relate those kinetics to variable stoichiometry and cell size, and not assume simple fixed relationships. For phosphate transport, as for ammonium transport, *TRD*_*max*_ is likely very much higher than *TRD*_*Gmax*_. In addition, the strongly curved form of the P:C quota relationship [57] will also have a strong impact upon *K*_*G*_ for P-limited growth as the P:C value in cells growing at *G*_*0*.*5*_ will be low.

### Motion (motility or sedimentation) effects on *K*_*G*_

Our analysis suggests that for smaller cells (ca. <5μm *ESD*) motion has little additional scope to moderate diffusion limitation. Above that size, the negative effect of size is greatly countered (though not negated) by motion through swimming or sedimentation (Figs 1–5). Note that sedimentation is affected directly by Stokes law; hence differences in cell mass between species, and with nutrient status may affect sedimentation rates [60]. While it may be tempting to explain motility primarily as a mechanism to enhance competitive advantage for nutrient transport (i.e., through lowering *K*_*G*_), the role of motility is also related to behaviour linked to vertical migration [61,62]. Motility is also important for finding prey to support mixotrophy, an activity present in even the very smallest flagellated species, with an *ESD* of <3 μm, *Micromonas* [63].

Sedimentation in diatoms is a common trait [64,65], often considered as detrimental but having clear advantages for nutrient acquisition at low concentrations in turbulent water systems (Fig 4). For diatoms, sedimentation adds significantly to the advantage of becoming increasingly vacuolate with larger *ESD* (Figs 4–7). Given that cell size usually also confers an anti-predator advantage, this means that larger diatoms appear better adapted to dominate in turbulent waters (in which their sedimentation de facto confers motility) than may otherwise appear.

### Maximum growth rate effects on *K*_*G*_

Our analysis indicates that the relationship between *G*_*max*_/*K*_*G*_ and *G*_*max*_ is flat for a given *ESD* (Fig 5). This relationship is useful as it permits the estimation of *K*_*G*_ for a given organism type, motility and size. It also means that a given organism will have a lower *K*_*G*_ if its *G*_*max*_ should decrease through adaptation, or indeed through acclimation, to different environmental conditions. The analysis also indicates that there is scope for a much greater spread in nutrient-related kinetics in larger cells (Fig 4). For smaller cells there is less effect of motility, and less variation in cell-C density; inter-species variation will thus generate increasing “noise” in the relationship between *ESD* and kinetics in larger cells.

The value of *G*_*max*_/*K*_*G*_ reflects many interactions and as a summary parameter provides an index for competitive advantage in simple nutrient-competition (bottom-up controlled) systems. The value of *K*_*G*_ itself also has important implications for the health of the cell; it defines the bulk water nutrient concentration (*S*_*∞*_) supporting a state of health aligning with *G*_*0*.*5*_. Health affects the intrinsic mortality rate of the cell, a factor that is typically not included in models scaled to nutrient status, but one that is important as a selective feature [66,67]. A poor health status adversely affects the operation of repair mechanisms, e.g. compensating for photo-damage [68], and explains the duration of the lag phase of growth seen when nutrient-starved microalgae are re-fed [69].

### Describing the relationship between nutrient concentration and growth rate

**Fig 9:**
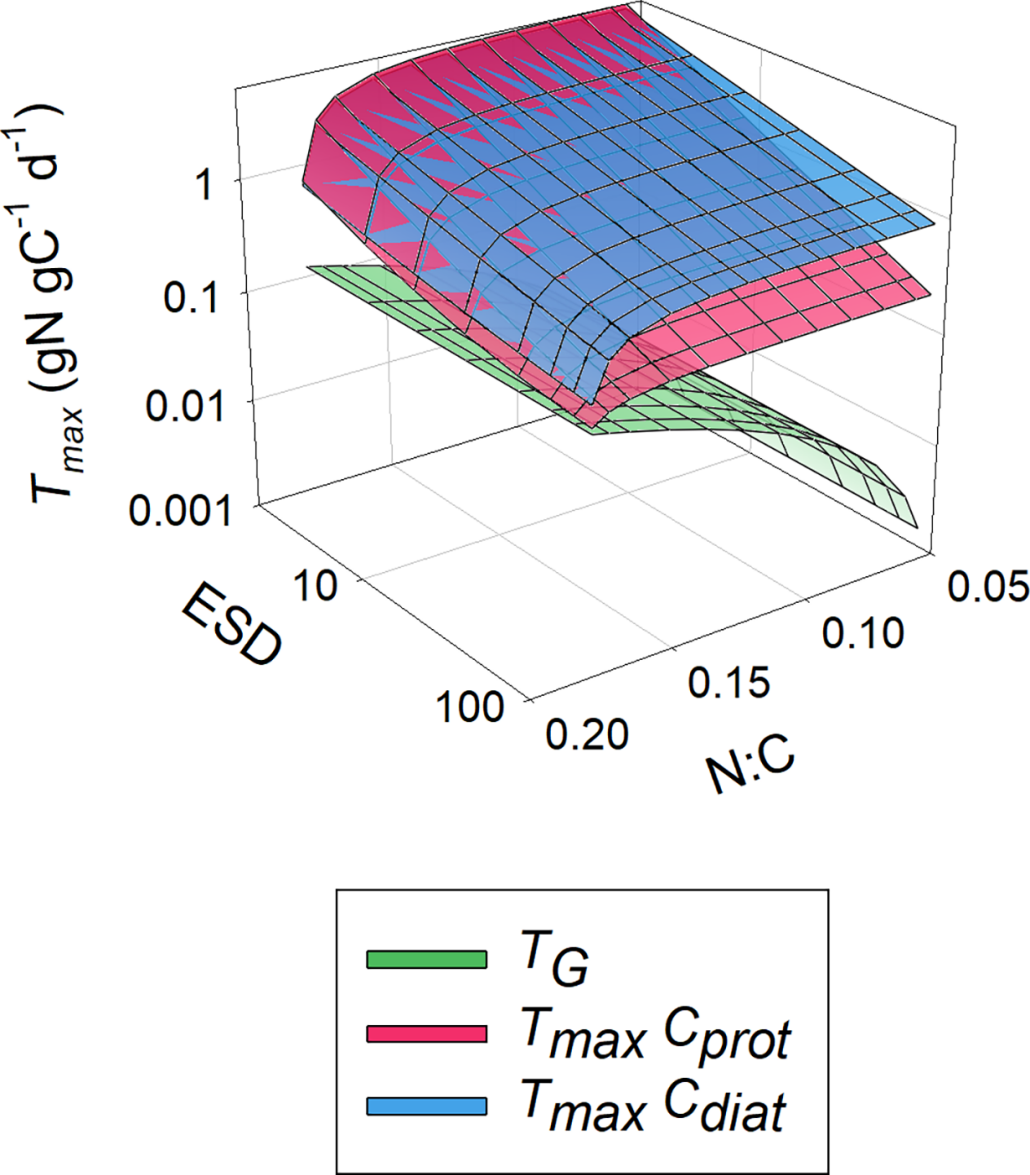
Relationship between *T*_*max*_ N-status (N:C) and cell size (*ESD*). This is shown for cells as protists or diatoms of different size (as equivalent spherical diameter, *ESD*), defined using Eqs 5, 6 and 7 with *KT*_*con*_ = 0.1. The green layer shows the transport need to support growth; the difference between this green layer and the potential transport rate *T*_*max*_ indicates the potential over-capacity for transport (see S2 Fig). The maximum growth rate was assumed as 0.693 d^-1^; at higher *G*_*max*_ the green layer is elevated there thus being less difference between *T*_*max*_ and the transport required to meet demand. Maximum and minimum N:C were assumed at 0.18 and 0.05 gN gC^-1^, respectively; the cellular carbon density was set via the allometric relationships for *C*_*prot*_ and *C*_*diat*_ [9]; the maximum transporter rate density was set at *TRD*_*max*_ = 0.4 pgN μm^-2^ d^-1^.

Simple models relate nutrient concentration to transport rate and thence to growth rate using a rectangular hyperbolic type 2 (RHt2) response curve, in line with Monod (1949) [70]. From our analysis (Figs 9, S1 and S3–S6) RHt2 cannot be expected to well define the actual relationship between nutrient concentration in the bulk medium (*S*_*∞*_) and transport. The fitting of RHT2 tends to over-estimate transport at lower nutrient availability and over or under estimate it at high availability. The expected relationship plateaus more abruptly than RHt2 can describe it. It is noteworthy that the fit of RHt2 to the modelled relationships was high (R^2^ > 0.95 in all instances, and most > 0.98); the “noise” in biological measurements that is inherent in experimental procedures of transport and growth rates [6] will inevitably result in a statistically acceptable fit to RHt2. Nonetheless, RHt2 does not appear to be appropriate, and the apparent subtle differences in the form of the described nutrient transport kinetics will manifest themselves in potentially important differences in competitive advantage in modelled populations. Such differences become more apparent when considering the form of the relationship between nutrient status and *T*_*max*_ (Fig 7), a topic that is also of consequence when describing the ammonium-nitrate interaction [71]. It is also important to couple nutrient-light limitations in the correct way, else the expected decrease in *K*_*G*_ with light limitation does not occur [72]. Interactions with temperature and allometry are also complex [53,73], with changes in cell size, overall growth rate, and differential impacts on transport vs metabolism [28,74]. All of this speaks to the importance of describing the relationship between multi-factor feedback interactions upon cell growth, with some attempt to simulate (de)repression of different metabolic pathways.

### Wider context & conclusions

In general, the importance and usefulness of using a single proxy as a determinant of competitive advantage seems overstated. This applies to usage of the value of *k*_*cat*_/*K*_*M*_ in enzyme kinetics, *U*_*max*_/*K*_*U*_ in studies of diffusion limitation, or *G*_*max*_/*K*_*G*_ in whole organism growth kinetics. Similarly, only considering stoichiometry represents too great a simplification in considerations of nutrient competition [59]. We simply have too limited knowledge of the real nutrient concentrations at the scales of consequence for these organisms (proximate to the cells), while we also know that factors such as alternative nutritional routes (nitrate vs ammonium vs dissolved organic -N; phosphate vs dissolved organic -P, phago-mixotrophy), different transporter types with different affinities for a given nutrient [14,16], allelopathy [75], palatability for grazers [76] and resistance to non-predator factors affecting cell mortality [77] are all important if not critical factors affecting competition at different times and places in the real world.

Our analysis, like many other studies, makes the unrealistic caveat of all-else-being-equal across a wide range of organism types, shapes, sizes, motilities and stoichiometries. So, while Fig 4 portrays a general theoretical pattern, application of that pattern to explain species competition for growth in the same water body must be viewed with extreme caution. It is of some comfort that the approach justifies (is consistent with) a common assumption that fast growing (r-select) species are disadvantaged in mature ecosystems where their slower growing (K-select) competitors have a better nutrient affinity (lower *K*_*G*_). However, simply relating *K*_*G*_ (or indeed any such parameter) to size is in any case highly problematic: many very small, non-motile cells tend to grow together (notably when P-stressed), and diatoms can grow in chains or mats, so that effective particle size (affecting boundary layer thickness and sedimentation) is often larger than it appears; the impacts of such changes are typically not included in models. Furthermore, little is known about interactions with alternative modes of nutrition, such as mixotrophy (including the use of dissolved organics), which likely vary significantly between organisms and will impact greatly upon the significance of *K*_*G*_ for a given limiting nutrient at any instant in time.

Within simple bottom-up controlled systems operating under non-steady-state conditions, possession of a higher growth rate is expected to endow a powerful competitive advantage under conditions of nutrient abundance. Larger growing cells need not be disadvantaged in such systems. However, smaller organisms appear always to be at an advantage for nutrient acquisition within nutrient limited systems running closer to steady-state, as epitomised by chemostat experimental systems and typically observed in the oligotrophic oceans. In a chemostat, at a given dilution rate the substrate concentration converges on that which enables the growth rate to match the dilution rate. Besides the logistic challenges in running chemostats to determine *K*_*G*_, there is also the real risk that the organisms adapt to enforced slow growth over many months [66]. It is notable that the predicted values of *K*_*G*_ from this study (Fig 2) are in the main very low, bordering on the level of chemical detection in the bulk media, even when assuming a transporter protein nutrient affinity *(K*_*T*_) of 1 μM. Interestingly, in modelled systems, the dynamics of the system may be more heavily controlled by the parameters controlling activity of zooplankton than by the value of *K*_*G*_ for phytoplankton [78]. It is also noteworthy that factors affecting cell size, motility/sedimentation, stoichiometry and cellular carbon density impact greatly upon predation kinetics and the value of the organism as food for the predator [79,26]. Thus, while motility enhances transport potential through decreasing boundary-layer limitations, motility is rather a double-edged-sword as it raises the likelihood of encountering a predator. For sure, simple comparisons between single-factors such as nutrient competition cannot possibly determine the true competitive advantage.

We can perhaps be more secure in considering the implications of our analysis for the evolution of an individual species, where intra-species competition is important. Here, within a particular cell line of a given species, the values of *K*_*G*_ and *G*_*max*_ can be expected to be linked; a faster growing cell will have a higher *K*_*G*_. This observation is consistent with a general feature of enzyme activity such that high *k*_*cat*_ is often associated with a high *K*_*M*_ [42], in consequence of a low *K*_*M*_ being deleterious for the rapid breakdown of the enzyme-substrate complex. Irrespective of species-species interactions, one may thus expect a trade-off between *K*_*G*_ and *G*_*max*_ and for this to be reflected in the evolution of a particular cell line. Taken alone, this is an important trade-off between traits affecting the benefit of fast growth and is consistent with the observation that cells forced to grow slowly in a low-dilution chemostat (noting that dilution rate = growth rate at steady-state, and that the residual nutrient concentration is lower at low dilution rates) evolve a lower *G*_*max*_ than the parent population [66]. The complexity of trade-offs in the evolution of individual enzymes [42] perhaps warns against attempting too-tight a linkage between *K*_*G*_ and *G*_*max*_ in terms of trait trade-off arguments at the whole organism level.

## Methods

In the following we assume that the transporter rate density (*TRD*) has a maximum possible value (*TRD*_*max*_); that is to say that, the plasma-membrane can only contain so-many nutrient transporter proteins over a given area. We assume *TRD*_*max*_ to be the experimentally determined maximum rate of 0.4 pgNμm^-2^ d^-1^ (from the diatom *Thalassiosira*, using experimentally computed C-cell; Table 2). Note, that the actual expressed value of *TRD*, and the instantaneous operation of transporter proteins, may be down-regulated due to long or short-term feedback linked to satiation and cellular nutrient status. It is assumed that all transporter proteins, contributing to *TRD*, have the same transport potential irrespective of the organism; hence we assume no features of the plasma-membrane or allied cell wall structure affect the functional value of *k*_*cat*_ or *K*_*T*_ of the embedded transporter proteins.

The value of *T*_*max*_ varies with the physiological status of the cells. Here we consider the N-status as indexed by the cellular N:C. The N-status is described as a normalised N:C quota [57] such that minimum stress is given by *NCu* = 1, and maximum stress by *NCu* = 0. The equation defining *NCu* is:

$$NCu=\frac{\left(1+KQ)∙(NC-{NC}_{min}\right)}{\left(NC-{NC}_{min})+KQ∙({NC}_{max}-{NC}_{min}\right)}$$ (4)

*NC* is the current cellular mass ratio of N:C, which ranges between *NC*_*min*_ when *G* is limited to 0 by supply of nutrient-N, and *NC*_*max*_ when *G* = *G*_*max*_. *KQ* is a curve shaping constant, which at a *KQ* = 10 gives the expected near-linear relationship between N:C and the growth rate, *G* [6].

The value of *T*_*max*_ can be derived experimentally (as in S2 Fig). *T*_*max*_ can alternatively be described hypothetically as increasing with decreasing nutrient status. To achieve the latter, here we use a simple curve form that carries a minimum of *G*_*max*_ × *NC*_*max*_ and rises rapidly as the N-status, *NCu*, decreases (i.e. as N:C decreases from *NC*_*max*_ to *NC*_*min*_). This equation contains a normalised RHt2 description which for values of 0≤*NCu*≤1 will return a value of 0 to 1 irrespective of the value of *KT*_*con*_, which is a curve setting constant (the lower the value the steeper the curve, increasing *T*_*max*_ as N:C decreases with N-stress).

$$T_{max}=G_{max}∙{NC}_{max}∙\left(1+T_{add}∙\frac{\left(1+{KT}_{con})∙(1-NCu\right)}{\left(1-NCu+{KT}_{con}\right)}\right)$$ (5)

The value of *T*_*add*_ provides a simple approach to reflect the diversity in scaling between the very highest expressed *T*_*max*_ and that required to support *G* = *G*_*max*_, as broadly seen in real organisms (S2 Fig). *T*_*add*_ acts as a multiplier for *T*_*max*_ (dimensionless); e.g. *T*_*add*_ = 1, will at *NCu* = 0 double the value of *T*_*max*_ over that expressed when *NCu* = 1 with *G* = *G*_*max*_. If *T*_*add*_ = 0, then Eq 5 describes a flat *T*_*max*_, as is *de facto* assumed in most models [72,80].

The maximum possible value of *T*_*add*_ in Eq 5 is a function of the value of *TRD*_*max*_ permitting us to explore the allometric and allied scaling of transport potential by reference to the maximum possible *TRD* (which here we consider as 0.4 pg nutrient-N μm^-2^ d^-1^) and also to the value of *TRD* required to support *G*_*max*_, *TRD*_*Gmax*_. From Eq 2, we obtain:

$${TRD}_{Gmax}=\frac{G_{max}∙{NC}_{max}∙C_{cell}}{SA}$$ (6)

*C*_*cell*_ is the C content per cell (pgC); this value as a function of *ESD* is described as per [9]. *SA* is the cell surface area (μm^2^), and *NC*_*max*_ is the mass N:C at *G* = *G*_*max*_.

*T*_*add*_ is then given by

$$T_{add}=\frac{{TRD}_{max}-{TRD}_{Gmax}}{{TRD}_{Gmax}}$$ (7)

From Fig 8, it can be seen how the value of *TRD*_*Gmax*_ varies between organism configurations, increasing with size and *G*_*max*_. In particular large protists with their high demands for nutrients become limited by the value of *TRD*_*max*_ at high growth rates, i.e. *TRD*_*Gmax*_ approaches the maximum density of 0.4 pg nutrient-N μm^-2^ d^-1^. Fig 6, for a hypothetical organism with a fixed cellular carbon density (*C*_*150*_), shows the potential for smaller organisms to have scope for a far higher excess transport capacity; that is *TRD*_*max*_: *TRD*_*Gmax*_ = δ_*TRD*_ is higher for small cells, and this excess is higher again at lower *G*_*max*_. However, in realty larger cells are less C-dense [9], and this is even more apparent for diatoms as these are relatively even more vacuolated; this mitigates against the simple allometric response (Fig 9; Cf. Fig 2).

From the value of *T*_*max*_, the transport rate (*T*) is given by Eq 8 (Cf. Eq 1), where *S*_*0*_ is the nutrient concentration at the plasma membrane surface, and *K*_*T*_ is the half saturation constant for the nutrient transporter protein,

$$T=T_{max}∙\frac{S_{0}}{S_{0}+K_{T}}$$ (8)

This is rearranged to obtain *S*_*0*_:

$$S_{0}=\frac{{TK}_{T}}{T_{max}-T}$$ (9)

In reality, the value of *T* is limited by diffusion at low nutrient concentrations. This limitation sets a relationship between *S*_*∞*_ and *S*_*0*_. From Eqs 16 and 17 in [25], developed from [35], the transport rate of nutrient into the cell (*T*, ng cell^-1^ d^-1^) is related to the gradient between the bulk nutrient concentration and the nutrient concentration at the cell surface (*S*_*∞*_*−S*_*0*_, ng L^-1^) via the following equation:

$$T=\frac{D}{r}(1+0.5∙\frac{r}{D}∙c)∙4\pi r^{2}(S_{\infty }-S_{0})$$ (10)

Here, *r* is the cell radius (μm), *D* is diffusivity (μm^2^ d^-1^), *c* is the organism’s speed of motion either due to swimming or sedimentation (μm d^-1^). The thickness of the boundary layer impacts upon the difference between *S*_*∞*_ and *S*_*0*_; the larger the cell, and the slower its motion through the water, the greater is the value of (*S*_*∞*_−*S*_*0*_) for a given value of *T*. By rearranging Eq 10, we obtain the value of *S*_*∞*_.

$$S_{\infty }=\frac{T}{4D\pi r(1+\frac{0.5rc}{D})}+S_{0}$$ (11)

Cell motility (*c*, μm s^-1^) was configured using an empirical allometric equation using data from Sommer 1988 [81] and Visser & Kiørboe 2006 [82] according to [79] as:

$$c=38.542*{\left(ESD\right)}^{0.5424}$$ (12)

Sedimentation rates (*c*^*sed*^; μm s^-1^) were computed using Stoke’s law, from the cell radius (*r*; μm), cell density (ρ_*org*_; assumed here to be 1.0634 kg L^-1^), seawater density (ρ_*w*_; assumed here to be 1.033 kg L^-1^), dynamic viscosity (*η*; assumed here to be 1.0846x 10^−3^ Pa s), and acceleration due to gravity (*g*; 9.8 m s^-2^).

$$c^{sed}=2gr^{2}\frac{\rho_{org}-\rho_{w}}{9\eta }$$ (13)

In order to compute the value of the bulk-water nutrient concentration (*S*_*∞*_) that supports a given growth rate, the above equations were constructed to enable organism size, allometric parameters and motility to be altered. For given values of *G*_*max*_, *NC*_*max*_ and *NC*_*min*_, the rate of N transport required to support a given *G* is computed. For a given cell size, cellular carbon density and N:C, we calculate the cell surface area, and the N-cell density at a given *G*. From these the rate of N-source transport per cell surface area is computed to support the given *G*; this is the value of *T* in Eqs 8 and 10.

## Supporting information

S1 Text Information on supplementary figures. (DOCX)

S1 Fig Schematic showing the theoretical relationship between substrate concentration at the site of the transporter. Shown is the activity of a single transporter protein (T1), with *k*_*cat*_ = 1 (units of transporter-specific activity per time) and half saturation *K*_*T*_ = 1 (units of substrate concentration at the transporter site), and the collective activity of 4, 8 or 16 of such transporter proteins within a cell plasma-membrane. Note that *K*_*T*_ remains the same, while the effective maximum transport rate (*T*_*max*_, as represented by the plateau value of the transport rate) is a product of *k*_*cat*_ and the number of transporter proteins. Consider now the instance where the organism can attain its maximum growth rate (*G*_*max*_) through a transport rate of T = 2 (marked by the line at T@ *G*_*max*_), then the substrate concentration that would support *G*_*0*.*5*_ (i.e., the value of *K*_*G*_) can be seen to be lower than *K*_*T*_ by a margin related to the number of transporter proteins. All units are arbitrary.
(TIF)

S2 Fig Values of *T*_*max*_ for the transport of ammonium or nitrate in *Emiliania huxleyi* and *Heterosigma carterae*. Increasing N-stress is indicated by the declining mass ratio of N:C. The grey line, labelled “Growth”, indicates the rate of N-transport required to support steady state growth rate at a given level of cellular N:C; this assumes that the growth rate relationship with N:C does not vary with nutrient source (there is no evidence to the contrary). Note how the value of *T*_*max*_ increases during initial N-stress and then decreases at extreme N-stress (i.e., at low N:C), that the ammonium curves are above those for nitrate, and that at high N:C the transport of nitrate is repressed below that required to support growth (i.e., the value of *T*_*max*_ declines below that indicated by the “Growth” curve) before the transport of ammonium. Curves recreated from the experimental data [18].
(TIF)

S3 Fig Relationship between N-source substrate concentration and N-specific transport rate for different protist sizes. Protists are considered of *ESD* 5, 20 or 60μm, with *G*_*max*_ = 0.693 d^-1^. The left-hand column of plots assumes the value of *T*_*max*_ increases with deteriorating N-status; *TRD*_*max*_ was assumed 0.4 pgN μm^-2^ d^-1^. The right-hand column of plots assumes *T*_*max*_ fixed in line with the transport rate required to support *G*_*max*_. The grey curve (“Ssur”) indicates the relationship at the membrane surface; this relationship would also apply if diffusion limitation was zero (or ignored). The solid black curve (“S 0M”) assumes no motility; the dashed black curve (“S M”) assumes motility as allometrically defined by Eq 12. The solid or dashed blue curves are for rectangular hyperbolic type 2 (RHt2) fits through the solid or dashed black curves (nonmotile vs motile, “S 0M RHt2” vs “S M RHt2”,respectively), with unconstrained fitted values of *T*_*max*_ and *K*_*T*_. The solid or dashed red curves are for rectangular hyperbolic type 2 (RHt2) fits through the solid or dashed black curves (nonmotile vs motile, “S 0M RHt2 fGmax” vs “S M RHt2 fGmax”, respectively), with unconstrained fitted values of *K*_*T*_. but with the fitted value of *T*_*max*_ constrained (fixed) to align with *G*_*max*_ (i.e., 0.693 d^-1^). Note the different x-axis ranges.
(TIF)

S4 Fig As S3 Fig but for diatoms. The dashed black curve assumes sedimentation as allometrically defined by Eq 13.
(TIF)

S5 Fig As S3 Fig, but for protists with *G*_*max*_ = 1.386 d^-1^. (TIF)

S6 Fig As S4 Fig, but for diatoms with *G*_*max*_ = 1.386 d^-1^. (TIF)
