# Supplementary material for: Effects of growth rate, cell size, motion, and elemental stoichiometry on nutrient transport kinetics
Source: PLoS Comput Biol. 2018 Apr 27;14(4):e1006118. doi: 10.1371/journal.pcbi.1006118 (PMC5942848; doi:10.1371/journal.pcbi.1006118)
Supplement: S1 Text — (DOCX) [file pcbi.1006118.s001.docx]

**S1 Text. Information on the supplementary figures.**

Figure S1 provides a schematic relating the hypothetical activity of individual transporters to that of the collective transporters in a cell, thus supporting its growth. From this is can be seen how the concentration of nutrient required to support half the maximum growth rate (*K_G_*) is smaller than the value of *K_T_*, and how this difference reflects the number of transporter protein molecules in the cell plasma-membrane. From Fig S1 it is also apparent that the relationship between the substrate concentration and growth rate *G* is not expected to conform to a RHt2 curve, but rather that it is truncated at higher values of substrate concentrations. Thus, a cell with the “T8” configuration (Fig S1) will show a relationship between substrate and growth rate that will truncate from a substrate value of ca. 0.25.

Changes in ammonium and nitrate transport potential with nutrient status are shown in Fig S2. For N-replete phytoplankton growing on ammonium, *T_max_* for nitrate will be zero as the ability to acquire nitrate is completely repressed. As N-stress develops, *T_max_* for ammonium transport increases rapidly, and an ability to transport nitrate develops (*T_max_* for nitrate >0). Evidence then suggests that at more extreme levels of N-stress these transporter capabilities decrease, perhaps in part reflecting internal cellular recycling of resources away from transporter proteins to more critical activities, so that *T_max_* for both ammonium and for nitrate decline. It is noteworthy that, similar to the value of *T_max_*, the need for the nutrient to support a given growth rate varies with nutrient status (as indicated by the grey “Growth” lines in Fig 2). The value of *T_max_* may be set as a constant in models (e.g. [80], defined to enable the maximum quota to be attained at maximum growth rate (e.g., for transport of N controlled by the N:C quota *NC*, *T_max_*= *µ_max_* x *NC_max_*). Rather than describing the (de)repression seen in reality (and as modelled by [7], see also [72]), control of transport may be made as an abrupt termination at the upper extreme of N-sufficiency, e.g. [83], to prevent transport into a cell that is already at its maximum nutrient quota (e.g., when N:C has attained *NC_max_* due to P-stress).

**References**

7. Flynn KJ, Fasham MJR, Hipkin CR. Modelling the interactions between ammonium and nitrate uptake in marine phytoplankton. Philos Trans R Soc B Biol Sci. 1997;352:1625–45.

72. Flynn KJ. Do we need complex mechanistic photoacclimation models for phytoplankton? Limnol Oceanogr. 2003;48(6):2243–9.

80. Geider RJ, MacIntyre HL, Kana TM. Dynamic model of phytoplankton growth and acclimation: Responses of the balanced growth rate and the chlorophyll a:carbon ratio to light, nutrient-limitation and temperature. Mar Ecol Prog Ser. 1997;148(1–3):187–200.

83. Pahlow M, Oschlies A. Chain model of phytoplankton P, N and light colimitation. Mar Ecol Prog Ser. 2009;376(2):69–83.
