# Supplementary figures and images for: Effects of growth rate, cell size, motion, and elemental stoichiometry on nutrient transport kinetics
Source: PLoS Comput Biol. 2018 Apr 27;14(4):e1006118. doi: 10.1371/journal.pcbi.1006118 (PMC5942848; doi:10.1371/journal.pcbi.1006118)

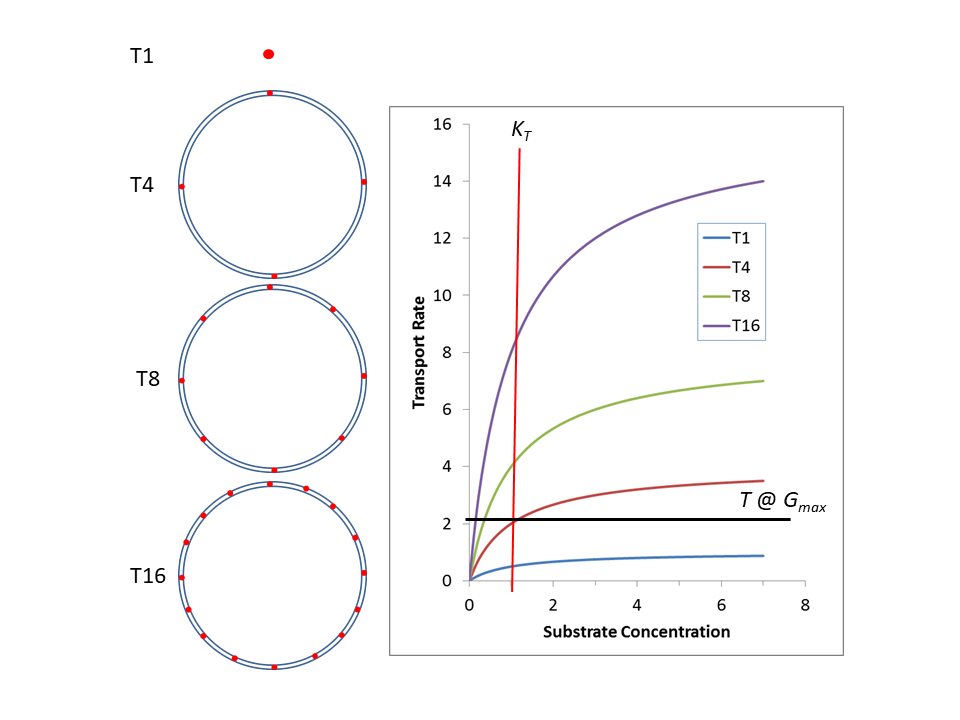

Supplement: S1 Fig — Shown is the activity of a single transporter protein (T1), with kcat = 1 (units of transporter-specific activity per time) and half saturation KT = 1 (units of substrate concentration at the transporter site), and the collective activity of 4, 8 or 16 of such transporter proteins within a cell plasma-membrane. Note that KT remains the same, while the effective maximum transport rate (Tmax, as represented by the plateau value of the transport rate) is a product of kcat and the number of transporter proteins. Consider now the instance where the organism can attain its maximum growth rate (Gmax) through a transport rate of T = 2 (marked by the line at T@ Gmax), then the substrate concentration that would support G0.5 (i.e., the value of KG) can be seen to be lower than KT by a margin related to the number of transporter proteins. All units are arbitrary. (TIF) [file pcbi.1006118.s002.tif]

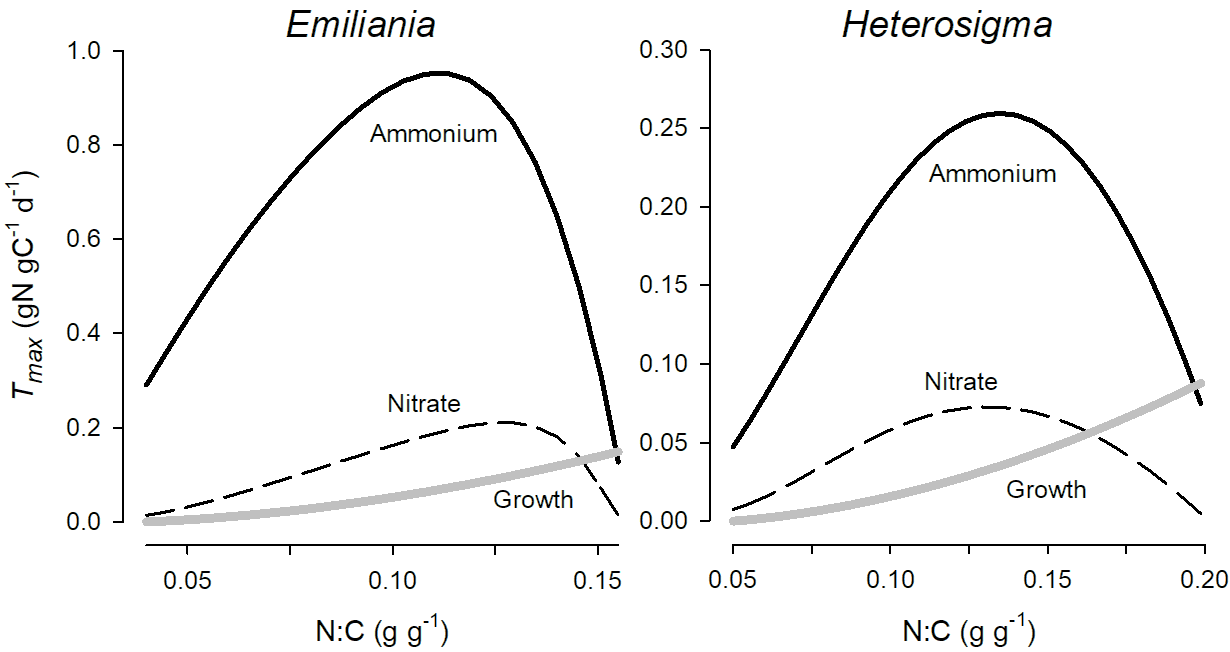

Supplement: S2 Fig — Increasing N-stress is indicated by the declining mass ratio of N:C. The grey line, labelled “Growth”, indicates the rate of N-transport required to support steady state growth rate at a given level of cellular N:C; this assumes that the growth rate relationship with N:C does not vary with nutrient source (there is no evidence to the contrary). Note how the value of Tmax increases during initial N-stress and then decreases at extreme N-stress (i.e., at low N:C), that the ammonium curves are above those for nitrate, and that at high N:C the transport of nitrate is repressed below that required to support growth (i.e., the value of Tmax declines below that indicated by the “Growth” curve) before the transport of ammonium. Curves recreated from the experimental data [18]. (TIF) [file pcbi.1006118.s003.TIF]

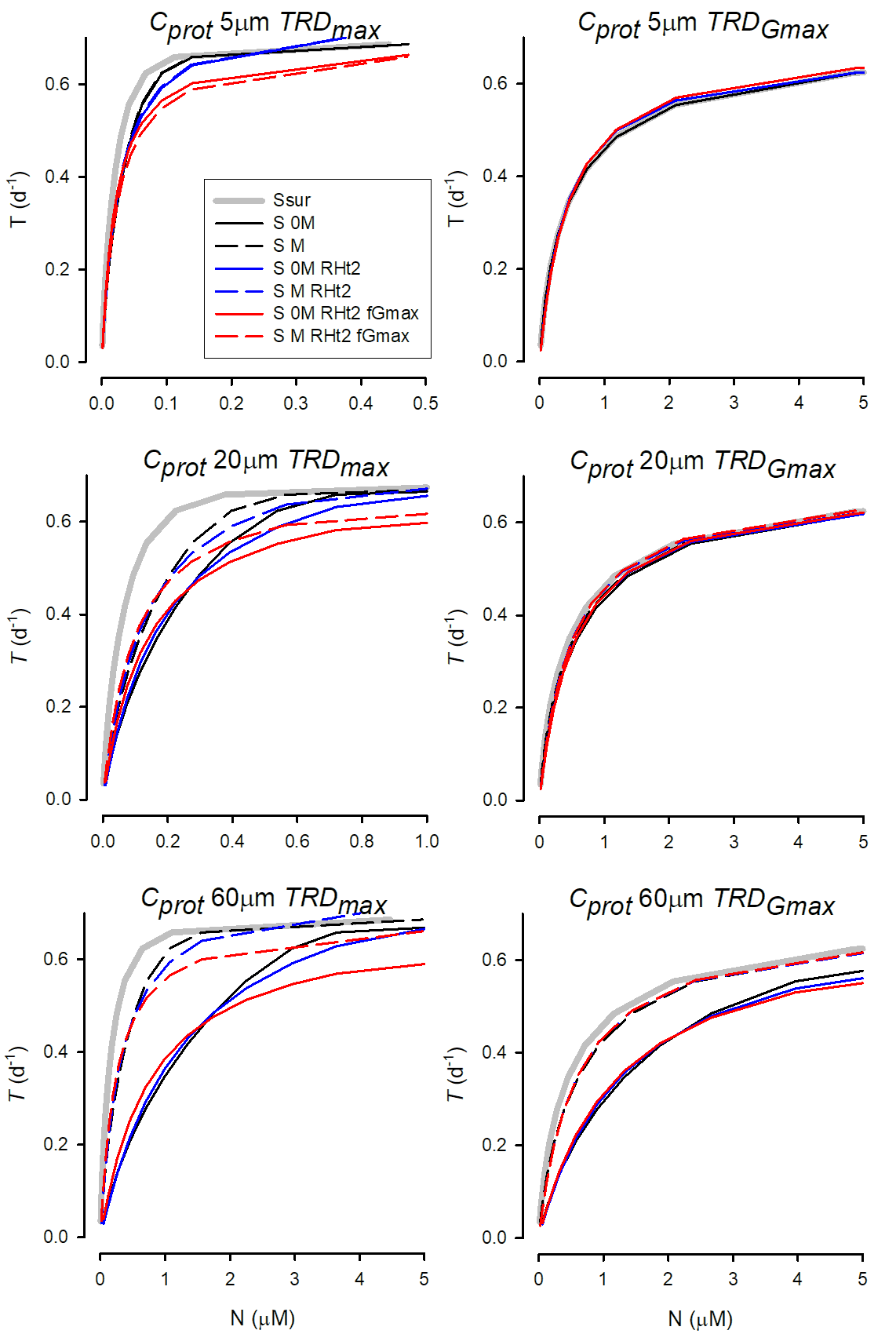

Supplement: S3 Fig — Protists are considered of ESD 5, 20 or 60μm, with Gmax = 0.693 d-1. The left-hand column of plots assumes the value of Tmax increases with deteriorating N-status; TRDmax was assumed 0.4 pgN μm-2 d-1. The right-hand column of plots assumes Tmax fixed in line with the transport rate required to support Gmax. The grey curve (“Ssur”) indicates the relationship at the membrane surface; this relationship would also apply if diffusion limitation was zero (or ignored). The solid black curve (“S 0M”) assumes no motility; the dashed black curve (“S M”) assumes motility as allometrically defined by Eq 12. The solid or dashed blue curves are for rectangular hyperbolic type 2 (RHt2) fits through the solid or dashed black curves (nonmotile vs motile, “S 0M RHt2” vs “S M RHt2”,respectively), with unconstrained fitted values of Tmax and KT. The solid or dashed red curves are for rectangular hyperbolic type 2 (RHt2) fits through the solid or dashed black curves (nonmotile vs motile, “S 0M RHt2 fGmax” vs “S M RHt2 fGmax”, respectively), with unconstrained fitted values of KT. but with the fitted value of Tmax constrained (fixed) to align with Gmax (i.e., 0.693 d-1). Note the different x-axis ranges. (TIF) [file pcbi.1006118.s004.TIF]

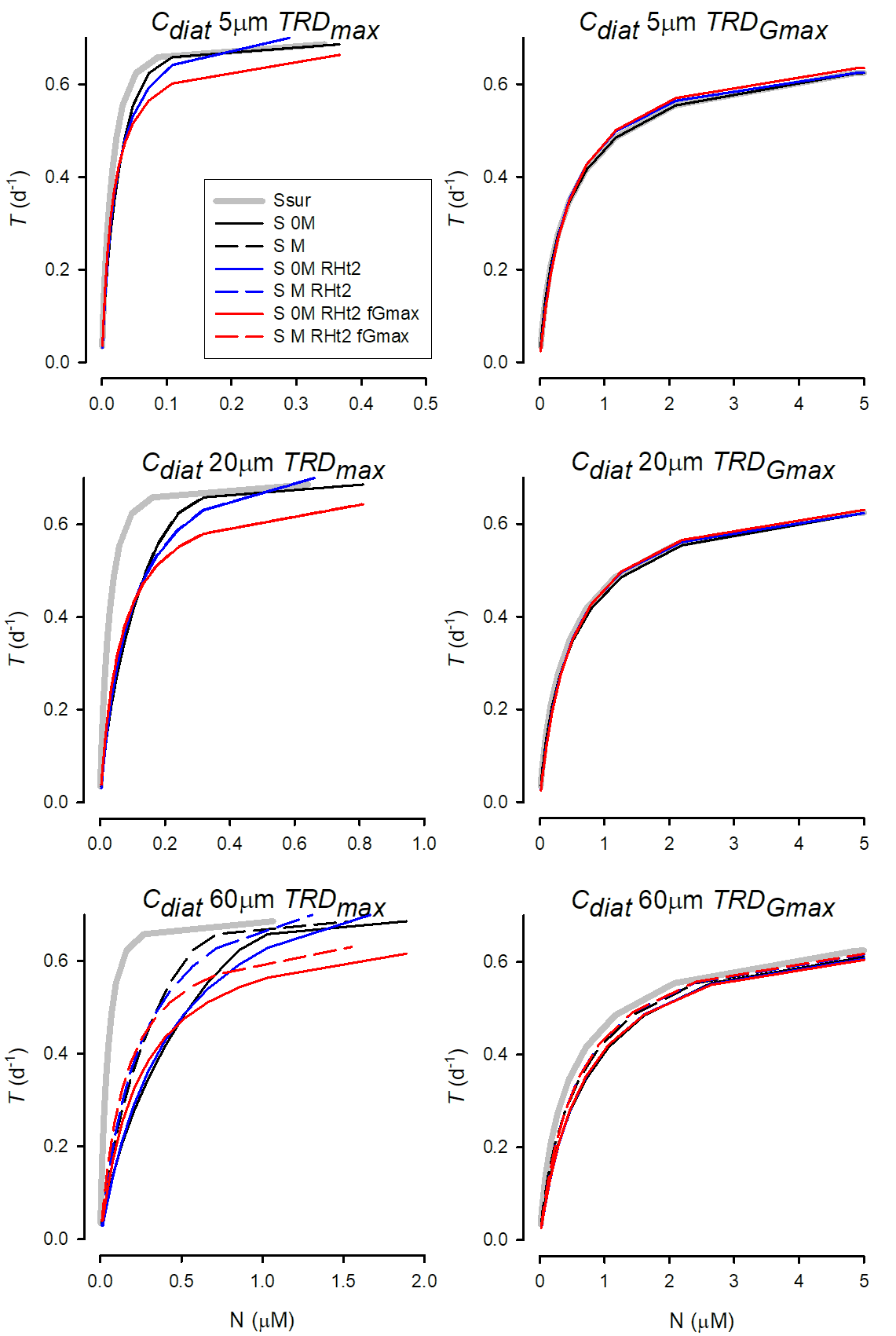

Supplement: S4 Fig — The dashed black curve assumes sedimentation as allometrically defined by Eq 13. (TIF) [file pcbi.1006118.s005.TIF]

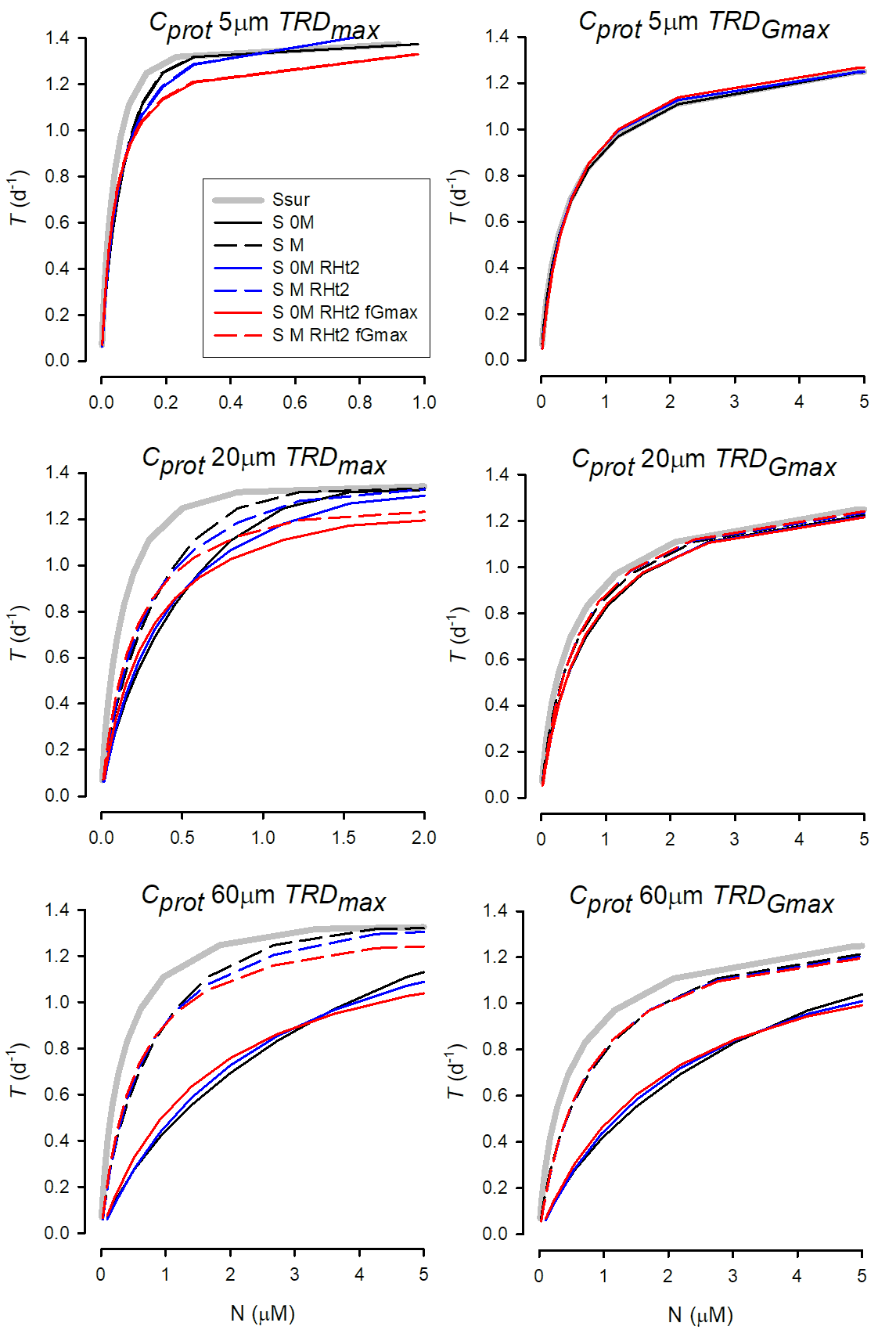

Supplement: S5 Fig — (TIF) [file pcbi.1006118.s006.TIF]

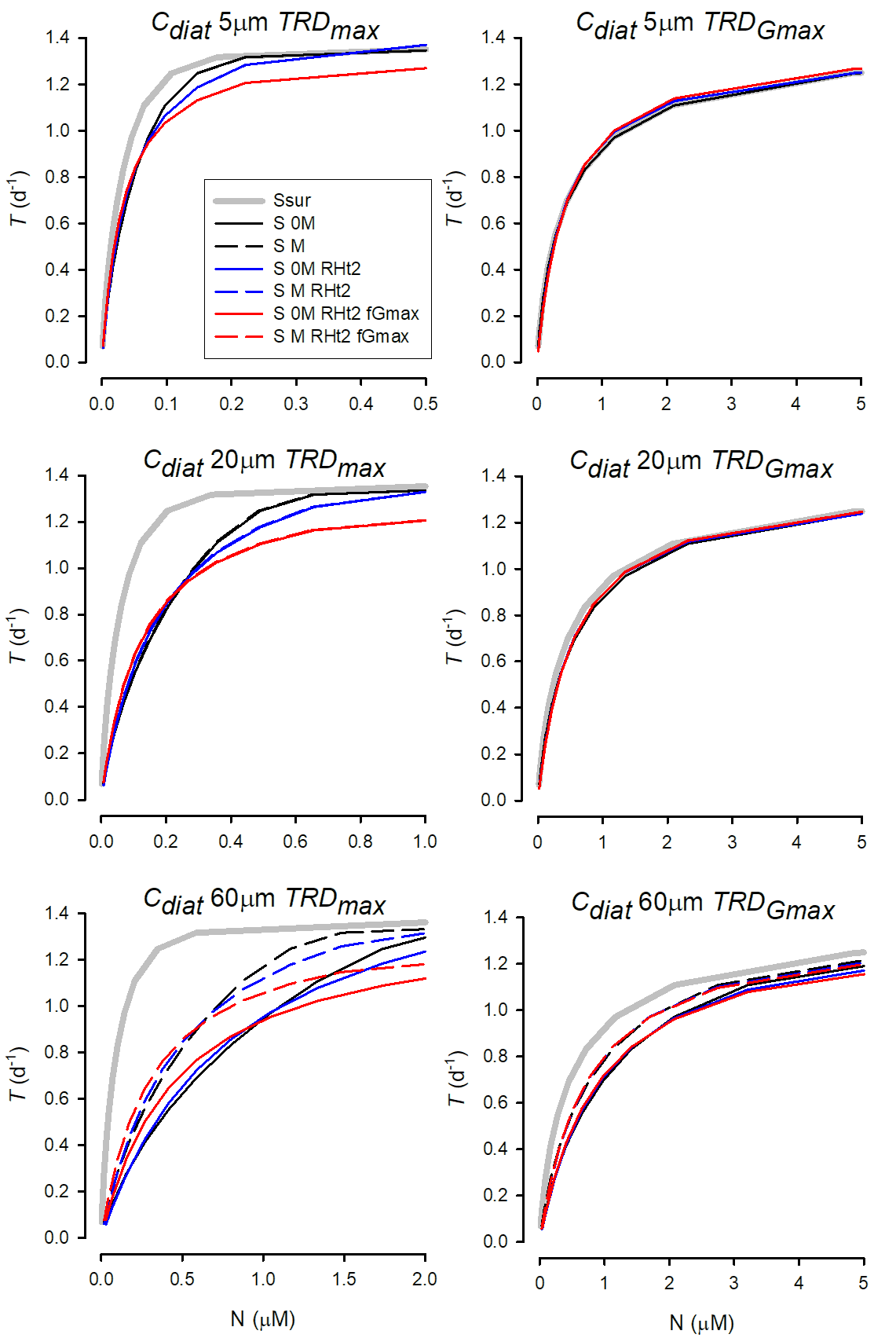

Supplement: S6 Fig — (TIF) [file pcbi.1006118.s007.TIF]
